# Modular approach for bimodal antibacterial surfaces combining photo-switchable activity and sustained biocidal release

**DOI:** 10.1038/s41598-017-05693-3

**Published:** 2017-07-12

**Authors:** Piersandro Pallavicini, Barbara Bassi, Giuseppe Chirico, Maddalena Collini, Giacomo Dacarro, Emiliano Fratini, Pietro Grisoli, Maddalena Patrini, Laura Sironi, Angelo Taglietti, Marcel Moritz, Ioritz Sorzabal-Bellido, Arturo Susarrey-Arce, Edward Latter, Alison J. Beckett, Ian A. Prior, Rasmita Raval, Yuri A. Diaz Fernandez

**Affiliations:** 1 Department of Chemistry and Centre for Health Technology, University of Pavia, Pavia, Italy; 2 Department of Physics, University Milano-Bicocca, Milano, Italy; 3 Department of Chemistry “Ugo Schiff” and CSGI, University of Florence, Florence, Italy; 4 Department of Pharmaceutical Sciences, University of Pavia, Pavia, Italy; 5 Department of Physics, University of Pavia, Pavia, Italy; 6 Open Innovation Hub for Antimicrobial Surfaces, University of Liverpool, Liverpool, UK; 7 Biomedical EM Unit, School of Biomedical Sciences, University of Liverpool, Liverpool, UK

## Abstract

Photo-responsive antibacterial surfaces combining both on-demand photo-switchable activity and sustained biocidal release were prepared using sequential chemical grafting of nano-objects with different geometries and functions. The multi-layered coating developed incorporates a monolayer of near-infrared active silica-coated gold nanostars (GNS) decorated by silver nanoparticles (AgNP). This modular approach also enables us to unravel static and photo-activated contributions to the overall antibacterial performance of the surfaces, demonstrating a remarkable synergy between these two mechanisms. Complementary microbiological and imaging evaluations on both planktonic and surface-attached bacteria provided new insights on these distinct but cooperative effects.

## Introduction

The development of smart on-demand antimicrobial surfaces has become a critical research area, addressing the issue of indiscriminate use of biocides implicated in the global emergence of antimicrobial resistance^1^. A significant challenge in this field is the need to combine on-demand antimicrobial functions at surfaces with static long-lasting effects. Static antibacterial surfaces are generally based on the release of metal cations with intrinsic biocidal properties^2^, e.g. sustained Ag^+^ release from surface-attached silver nanoparticles^3–5^. In this area we have recently demonstrated that after 24 h such surfaces can reduce the colony forming units (CFU) of planktonic *Escherichia coli* (*E*. *coli*) and *Staphylococcus aureus* (*S*. *aureus*) by 5–7 orders of magnitude^4^ and reduce the surviving fraction of *Staphylococcus epidermidis* (*S*. *epidermidis*) biofilms by 5 orders of magnitude^5^.

Alternatively, the use of light-activated antimicrobial surfaces provides a route towards responsive systems that can be triggered remotely and on-demand^6^. Translation of such technology to *in vivo* applications (e.g. on the surfaces of prostheses and implants) needs to be restricted to systems responding in the narrow near-infrared (NIR) spectral window (750–900 nm) in which water and living tissues are transparent and, therefore, not susceptible to damage^7–11^. In this context, photothermal conversion by non-spherical plasmonic nanoparticles (e.g. nanorods^12^ or nanostars)^7^ is becoming particularly interesting, allowing the use of local hyperthermia activated by NIR radiation to kill bacteria. In 2014, the first example of combined use of biocidal metal cations and photothermal conversion of light was published^13^, using biomimetically-coated Au/Ag core/shell nanorods, and demonstrating a synergistic antibacterial effect against planktonic *E*. *coli* and *S*. *epidermidis* bacteria upon NIR irradiation. More recently, we have developed a different approach, specially designed for surfaces, where glass substrates bearing monolayers of non-coated Ag nanoplates^14^ or Ag nanotriangles^15^ displayed antibacterial effects significantly enhanced upon NIR irradiation with respect to the intrinsic biocidal effects due to the release of Ag^+^ cations. Remarkably, the amount of Ag^+^ released from these surfaces was similar with and without NIR irradiation, leading us to hypothesise synergistic interactions between the photothermal response and the chemical microbicidal action^14^. However, in those systems the intrinsic contributions of thermal and chemical actions could not be unravelled.

In this work, we present a new modular approach that integrates, directly at a surface and in an orthogonal fashion, photothermally active gold nanostars (GNS)^7^ and chemical release of Ag^+^ from spherical silver nanoparticles (AgNP), retaining the distinct functionalities of the individual components.

**Figure 1:**
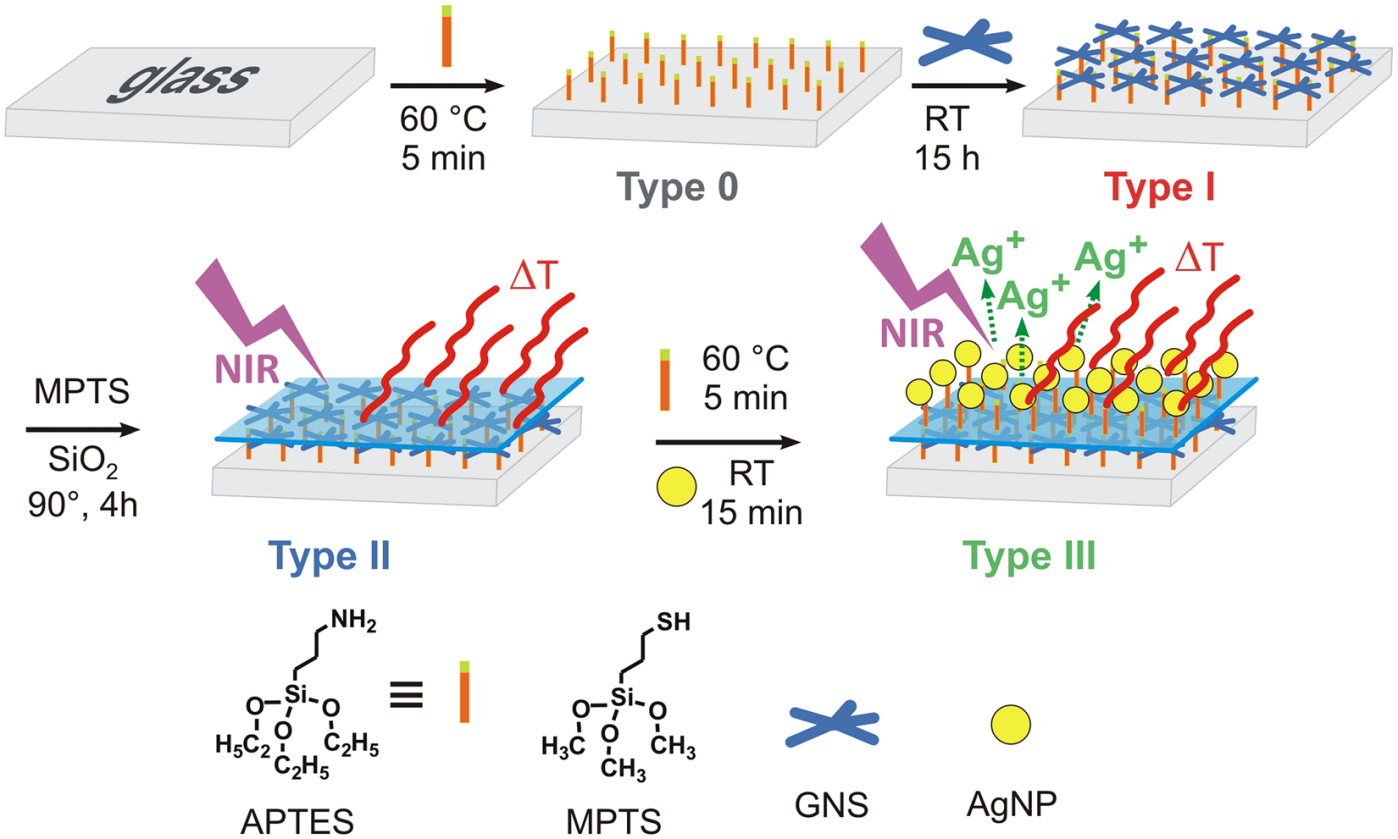
Synthetic route for the preparation of Type I-III surfaces (experimental details are described in the Methods section and in the Supporting Information). Type 0: amino-functionalised glass. Type I: gold nanostars (GNS) monolayer on glass. Type II: SiO_2_-coated GNS monolayer. Type III: Silver nanoparticles (AgNP) decorated surface with underlying monolayer of GNS.

This multi-layered system was constructed using the supramolecular chemistry concept of modular building blocks. Here, Type I surface (Fig. 1) is the photo-thermal unit, containing a monolayer of GNS grafted on APTES-functionalised glass. We subsequently introduced a spacer unit in the form of a SiO_2_ overlayer to obtain Type II surfaces (Fig. 1). This kind of surfaces may be a significant progress in the field of biomaterials, as it features a mechanically stable, chemically insulated, and NIR-responsive “heater” element, while displaying a bio-compatible interface with chemical and physical properties close to pristine glass, providing a versatile platform for more advanced architectures based on well-established alkoxysilanes and chlorosilanes chemistries^16, 17^. To demonstrate the versatility of this approach, we have grafted on Type II surfaces a monolayer of (3-Aminopropyl)triethoxysilane (APTES) and immobilised spherical AgNP to obtain Type III surfaces (Fig. 1). These multifunctional surfaces combine the long term biocidal effect of sustained Ag^+^ release with a flash antibacterial action activated on demand by NIR radiation. Comparison of the effects of Type II and Type III surfaces on both planktonic and surface-attached *S*. *aureus* and *E*. *coli* bacteria allowed us to unravel, for the first time, the distinct contributions of photo-thermal responses and AgNPs, demonstrating a remarkable synergistic effect between these two processes.

## Results and Discussion

### Synthesis of Type I-Type III surfaces

**Figure 2:**
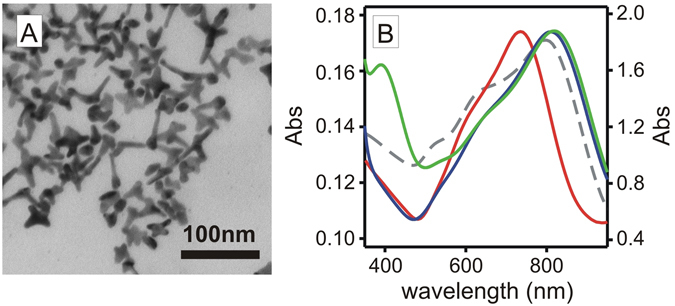
(**A**) TEM image of the GNS used in this work. (**B**) absorption spectra for GNS colloidal solution (grey dashed); Type I slides (red); Type II (blue); Type III (green).

The four different surface types described in Fig. 1 were prepared following a modular layer-by-layer approach. The further experimental details for the synthesis and characterisation of these surfaces are described in Methods and in the Electronic Supporting Information (ESI). The first step of the process was the functionalisation of microscopy glass coverslips (2.1 × 2.6 cm) with APTES (Type 0 slides) following protocols reported before^5, 18^, then GNS were grafted to obtain Type I surfaces. The GNS used in this work were obtained by seed-growth synthesis in water, using the weakly interacting surfactant Laurylsulphobetaine (LSB) as preferential-growth directing agent^19^. Under our synthetic conditions (see experimental details in ESI), deep-blue coloured colloidal solutions were obtained, containing prevalently penta-twinned five-branched GNS alongside a minority of monocrystalline 4-branched GNS (Fig. 2A **)**. Such solutions displayed a broad light absorption band in the 600–900 nm range, with λ_max_ at 800 nm (grey dashed line in Fig. 2B), corresponding to the Localised Surface Plasmon Resonance (LSPR) of the penta-twinned GNS^19^. The pH of these solutions was ~5.5, due to excess ascorbic acid used in the seed-growth process. Glass|APTES|GNS blue-coloured Type I surfaces were prepared by dipping Type 0 slides into the GNS colloidal solutions, exploiting electrostatic interactions between negatively charged GNS (Z = −12 mV) and positively charged amino-groups of grafted APTES molecules, protonated at the acidic pH of the GNS solutions. The quantity of grafted GNS was controlled by setting the dipping time, showing an increase of Abs vs time in the UV-Vis-NIR spectra of Type I surfaces (SI4, ESI), and confirmed by quantitative analysis of grafted gold and silver (SI4, ESI). Maximum surface coverage was reached after 3–6 h, but to ensure saturation we adopted 15 h dipping time as a standard. After such dipping time, the Au surface concentrations were determined by GNS oxidation with aqua regia followed by ICP-OES analysis, finding a surface concentration of 3.0 (±0.5) μg Au/cm^2^. It must be stressed that Ag^+^ is used in the seed-growth process and Ag(0) is contained within the GNS lattice in a 8–9% atomic percent^7^. Quantitative analysis of Type I slides confirmed the presence of Ag with surface concentration 0.32 (±0.03) μg Ag/cm^2^.

Anchoring GNS on APTES-functionalised glass led a LSPR blue shift (Δλ = −58 ± 9 nm) from the colloidal solution to Type I monolayers (red line in Fig. 2), attributed to the decrease in local refractive index (n) from water (n = 1.3339) to air (n = 1.0003) at the glass-air interface^7, 20^. To obtain Type II slides we first created a monolayer of mercaptopropyltrimethoxysilane (MPTS) on the exposed gold surface of the GNS in Type I surfaces, obtaining glass|APTES|GNS|MPTS. This coating gave a small red shift of the LSPR, with Δλ = +8(±2) nm (spectrum not shown in Fig. 1). The –Si(OCH_3_)_3_ groups of MPTS are reactive towards Na_4_SiO_4_ solutions, forming a SiO_2_ overlayer according to well established methods^21^ (see Experimental section in ESI for details), and allowing us to obtain glass|APTES|GNS|MPTS|SiO_2_ samples, i.e. Type II surfaces in Fig. 1. On Type II surfaces the SiO_2_ overcoating produced a significant red shift of the LSPR band (Δλ = 60 ± 15 nm, blue line in Fig. 2B) due to the local n increase (n = 1.4585 for SiO_2_). The thickness of the SiO_2_ coating depended on the dipping time in the Na_4_SiO_4_ solutions^21^ and in our case it was tuned to obtain a ~4 nm SiO_2_ layer, as estimated by AFM (SI8, ESI) and SEM imaging (SI9, ESI). This SiO_2_ overlayer on Type II surfaces dramatically increases the mechanical stability of the GNS monolayer, with respect to Type I surfaces. While the uncoated GNS monolayer on Type I surfaces is extremely fragile and can be removed even by gently rubbing the surface with a cotton stick, Type II surfaces are not easily scratched. Additionally, dipping Type II surfaces in aqueous solutions at pH = 2 or pH = 12 for 1 h under sonication did not cause any significant modification of the absorption spectrum of the grafted GNS. On the other hand, while Type I slides release traces of Ag^+^ in water from the silver contained within the GNS lattice, Ag^+^ release from Type II was not detected. All these facts evidence the increased robustness of Type II surfaces both in chemical and physical terms. Remarkably, the photothermal response of Type I and Type II slides (discussed in the next section) is identical, suggesting that Type II can be considered as a pristine glass surface equipped with an underlying GNS monolayer that acts as a chemically and physically segregated photothermal heater.

In agreement with this observation, we were able to graft an APTES monolayer on Type II surfaces with the same procedure used for the starting plain glass coverslips (see Methods and SI1, ESI) to obtain glass|APTES|GNS|MPTS|SiO_2_|APTES surfaces. We then functionalised these surfaces with citrate-capped spherical AgNP (d = 9 nm), according to literature methods^22^ and obtained Type III surfaces (glass|APTES|GNS|MPTS|SiO_2_| APTES|AgNP). The absorption spectrum of Type III (green line, Fig. 2B) showed the LSPR band of AgNP at 394 nm^4, 5^, alongside with the NIR band of the GNS, confirming successful incorporation of both nano-objects at the surface. Interestingly, the addition of AgNP did not modify the GNS LSPR. Large shifts and shape variations in LSPR bands at short inter-particle distances (<10 nm) have been predicted for spherical AgNP^23^ and rod-like Au nanoparticles^24^. This effect was not observed in our system, despite the relatively thin spacing created by the SiO_2_ interlayer. We attribute this to the different nature of the two types of nano-objects used (i.e. small Ag spheres vs large Au branched objects) which has been shown to induce poor plasmon resonance hybridization and negligible spectral changes^24^. In addition, our modular architecture, which segregates the two types of nano-objects into distinct layers, may also prevent efficient plasmonic coupling, preserving the NIR properties of the GNS.

**Figure 3:**
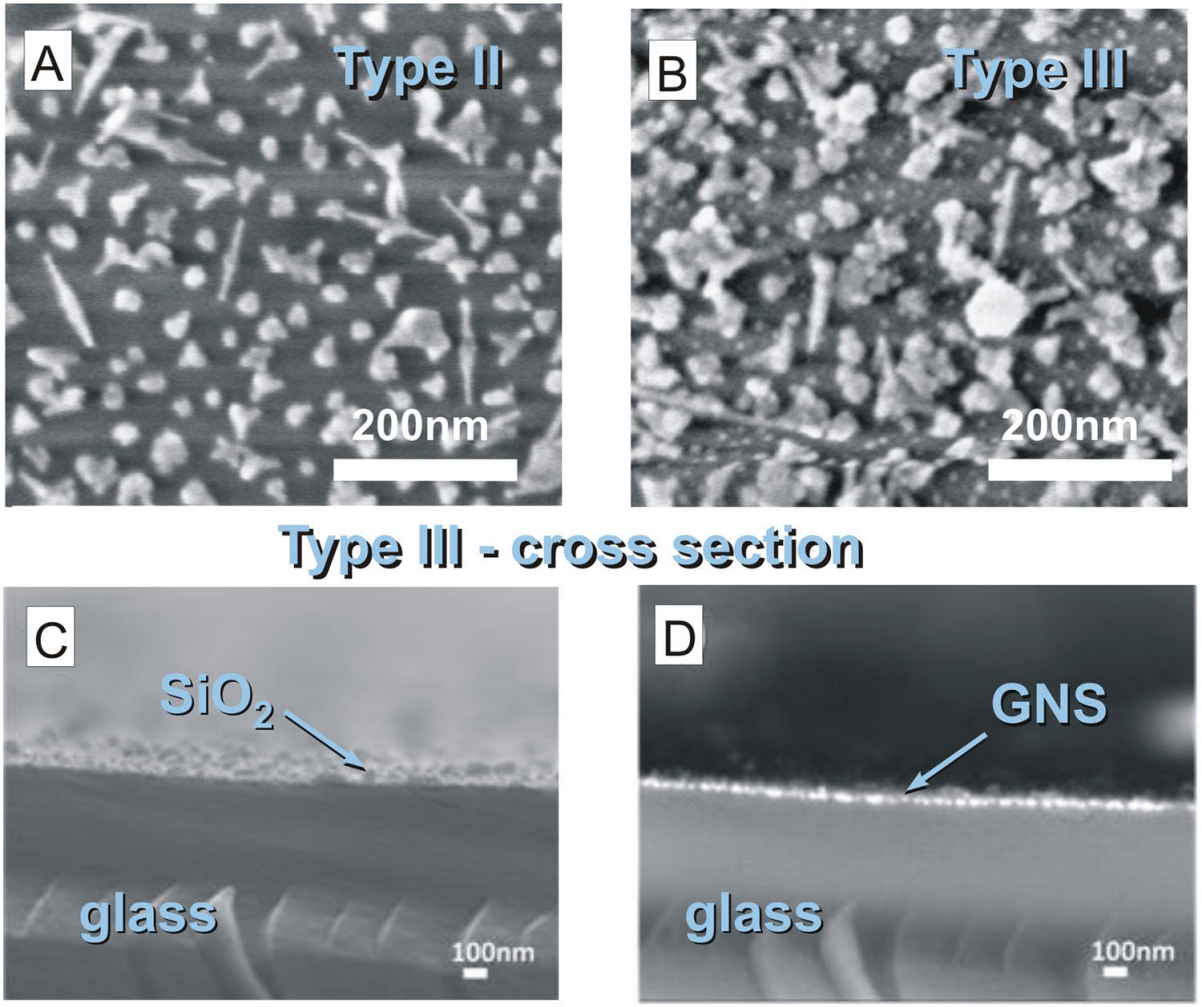
(**A**) HR-SEM image of Type II surfaces showing the morphology of GNS attached to the surface; (**B**) Type III surfaces showing AgNPs as small bright dots on top of the GNS monolayer. (**C**,**D**) SEM imaging of Type III slide cross-section showing (**C**) secondary electron detector topographic image and (**D**) backscattering electron detector (SEM BSD) image, with bright spots corresponding to the GNS monolayer (The sample is oriented upwards, from bottom to top: glass, GNS, SiO_2_, AgNP).

Contact angle measurements (SI7, ESI) were consistent with the modular construction and the layering sequence. SEM (scanning electron microscopy) also confirmed the grafting of small AgNP on Type III surfaces (Figs 3B and SI6) that are not observed in Type II samples (Figs 3A and SI6). SEM BSD (backscattered electron detection) allowed direct observation of the electron-dense GNS monolayer within the cross-section of Type III surfaces (Fig. 3C). The GNS appeared in the SEM BSD images as bright spots located at the sample surface and inserted within the multilayer structure (Fig. 3D). In the cross-section view, the increased roughness of the SiO_2_ could be also observed, while AgNP were too small to be imaged.

### Photothermal response of Type I, Type II and Type III surfaces

**Figure 4:**
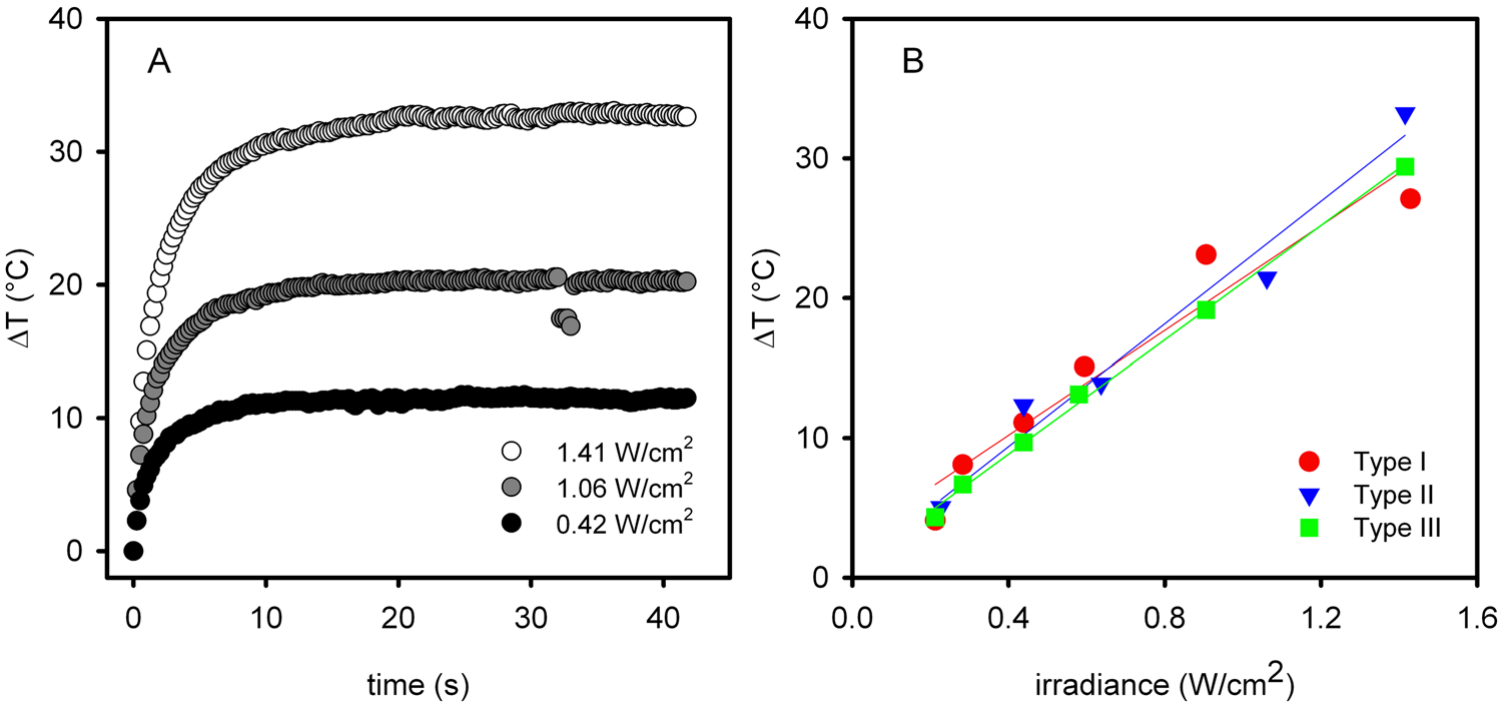
(**A**) Thermograms (ΔT vs time) for Type III surface irradiated with 800 nm laser, measured at different irradiance values (irradiance calculated considering a circular beam cross section, with a 3 mm beam waist). (**B**) stationary ΔT vs irradiance for Type I-III surfaces (solid lines are linear regressions).

The specific architecture of our modular multi-layered surfaces, spaced by a Vis-NIR transparent SiO_2_ interlayer, preserves the photo-thermal response of the GSN monolayer upon NIR irradiation (800 nm). By using a continuous laser source tuned on the LSPR maximum and delivering variable irradiances (0.2–1.4 W/cm^2^), we have tested Type I, II and III samples, containing the same GNS surface concentration. Upon laser irradiation, the surfaces experienced a time-dependent temperature increase that was directly measured using a thermocamera^25^. The thermogram profiles are characterised by a sharp temperature increase, followed by a plateau, as reported for Type III surfaces in Fig. 4A. Similar thermograms profiles were obtained for Type I and Type II surfaces (not shown). The observed T vs time behaviour can be rationalised considering the balance between photo-thermal conversion of incident light and heat dissipation from the surface (see ESI SI11 for physical model details). Maximum stationary temperatures were reached within 10 s, and the absolute increase (ΔT = T_plateau_ − T_0_) varied from 5 to ~35 °C at the different laser power densities examined. Figure 4B reports the stationary ΔT vs irradiance values for Type I, II and III surfaces (symbols) and their linear regressions (lines), showing that there is no significant difference in the photothermal response for the three types of surfaces.

### Noble metal content and release from the surfaces

The total amount of gold and silver in Type I-III surfaces was determined by full oxidation with aqua regia and ICP-OES quantitative analysis. The values obtained are reported in Table 1. As expected, the Au quantity did not significantly change in the three surfaces, with differences below the standard deviations. The quantity of Ag was identical in Type I and II (Ag in the lattice of the GNS), but increased for Type III, due to the additional AgNP layer. The quantity of silver in the spherical AgNPs grafted on the top layer of Type III surfaces can be directly determined by the difference in Ag content between Type III and Type II surfaces (0.6 μg/cm^2^). This value is comparable to the silver concentration from AgNP grafted on APTES-functionalised flat glass (0.73 μg/cm^2^)^5^. Considering that the grafted AgNP had an average diameter of 9 nm^5^ the AgNP surface density of 1.5 × 10^11^ AgNP/cm^2^ was calculated, in agreement with the number of AgNP per surface unit observed by HR-SEM (SI7, ESI).

**Table 1:** Total content and released fraction of metals on Type I, II, and III **s**urfaces.

	Total Au (µg/cm^2^)^a^	Total Ag (µg/cm^2^)^a^	Released Ag^+^ (µg/cm^2^)^a^			
			0.5 h	0.5 h laser	5 h	24 h
Type I	3.0 (0.5)	0.32 (0.03)	—^b^	—^b^	0.026 (0.003)	0.028 (0.003)
Type II	2.7 (0.3)	0.30 (0.03)	—^b^	—^b^	—^b^	—^b^
Type III	2.8 (0.3)	0.9 (0.1)	0.04 (0.02)	0.05 (0.02)	0.18 (0.02)	0.21 (0.01)
	After aqua regia		In contact with deionized water			

(see also SI10).

^a^Average values from at least three samples with standard deviations reported between brackets; ^b^Under detection limit.

We measured by ICP-OES the amount of Ag^+^ released from the surfaces into deionized water at different contact times, following established protocols^26^. The results, summarized in Table 1, show that a significant amount of Ag^+^ was released from Type III surfaces after 5 h and 24 h (0.18 μg/cm^2^ and 0.21 μg/cm^2^, respectively; the amount of Ag^+^ released at longer times did not increase significantly, see ESI SI10, in agreement with the Ag^+^ release profile already observed for AgNP monolayers on APTES^5^ and MPTS^4^). The Ag^+^ released after 5 h and 24 h corresponds to 30% and 35% of the total silver content in the AgNP overlayer, respectively. Conversely, the Ag released from SiO_2_-coated Type II surfaces after 24 h was under ICP-OES detection limit, and Type I surfaces released only small amounts of Ag^+^ after 24 h.

It is important to highlight that at short contact times (30 min) for Type III surfaces only small quantities of Ag^+^ were released, while on Type II surfaces no significant release was detected. Remarkably, the amount of Ag^+^ released after 30 min was not modified upon laser irradiation. These results suggest that within our modular approach, each surface functionality preserves its intrinsic property in an orthogonal fashion, i.e. the GNS deliver their inherent photo-thermal activity upon near-infrared irradiation, while AgNP release certain Ag^+^ concentration, irrespectively of light activation.

### Antimicrobial activity on planktonic bacteria

We investigated the antimicrobial activity of these surfaces on planktonic bacteria focusing on two distinctive effects: (a) the static release of Ag^+^ and (b) the photothermal response upon irradiation. First, the intrinsic antimicrobial activity of Type III surfaces against *E*. *coli* and *S*. *aureus* was investigated in absence of irradiation, to elucidate the static chemical contribution of Ag^+^ release. Previously, we reported an experimental protocol to evaluate the microbicidal effect (ME) within thin liquid films in contact with functionalized surfaces, using the expression^4^:

1 $${\rm{ME}}=\,\mathrm{log}\,{\rm{NC}}-\,\mathrm{log}\,{\rm{NE}}$$

where NC and NE are the number of colony forming units (CFU/mL) developed on non-functionalised control glass and on active surfaces, respectively. For Type III slides, after contact times of 5 h and 24 h, a significant bactericidal effect was observed (Table 2), as expected from the presence of the AgNPs overlayer on these surfaces. Longer contact times (24 h vs 5 h) are needed to obtain larger ME values, and the effects are stronger against *E*. *coli* with respect to *S*. *aureus*, in agreement with the literature^2, 4^. Control experiments with Type II slides (SiO_2_ covered GNS) showed no ME after 5 h and 24 h for both bacterial strains investigated, indicating that this material behaves as pristine glass if no irradiation is applied.

**Table 2:** Microbicidal effect on planktonic bacteria for non-irradiated surfaces (ME) and after laser irradiation (TME).

	ME^a^		TME^a^	
	(Type III) 5 h	(Type III) 24 h	(Type II) 0.5 h	(Type III) 0.5 h
*E*. *coli*	1.7 (0.1)	5.4 (0.3)	1.6 (0.2)	>6.0^b^
*S*. *aureus*	0.7 (0.2)	1.6 (0.2)	1.2 (0.1)	>6.0^b^
	Static Ag^+^ release		Photothermal	Combined

^a^Values are the average of three measurements. Standard deviations between brackets. ^b^A value higher than 6 indicates that no survived bacteria were found after the test.

The microbicidal effect exerted on planktonic bacteria by photothermal action upon NIR irradiation was then investigated for Type II and Type III samples. Surface irradiation was carried out for 0.5 h with an 800 nm continuous laser source (irradiance 0.25 W/cm^2^, ΔT = +5 °C measured at the air interface for all surfaces). In this case, the photo-thermal microbicidal effect (TME) was calculated as:

2 $${\rm{TME}}=\,\mathrm{log}\,{\rm{NCM}}-\,\mathrm{log}\,{\rm{NT}}$$

where NT and NCM are the CFU/mL developed on the modified glasses with and without laser irradiation, respectively. When Type II slides were used, we exclusively observed the effect of local photo-thermal action, as no Ag^+^ is released from these surfaces. At contact-irradiation times as short as 0.5 h, Type II slides showed measurable TME values (1.2 and 1.6 for *S*. *aureus* and *E*.*coli*, respectively; Table 2). Irradiation control experiments performed using unmodified glass showed no TME, demonstrating that the photothermal effect arises from the GNS and that direct laser-radiation damage is not significant under our experimental conditions.

Remarkably, on Type III slides, after just 0.5 h of irradiation, considerably higher TME values were observed for both *S*. *aureus* and *E*. *coli* (Table 2, TME >6 meaning more than 99.9999% bacteria killed). This is an impressive effect, considering that: (i) for Type II and Type III surfaces the temperature increase upon NIR irradiation is the same; (ii) the amount of Ag^+^ released from Type III surface after 0.5 h, with and without laser irradiation, is the same and very low; (iii) control experiments with no irradiation and 0.5 h contact time on Type III surfaces (plain glass as reference) disclosed negligible microbicidal effect. Therefore, the combination of photo-thermal activation and Ag^+^ release on Type III surfaces leads to enhanced antimicrobial responses, by orders of magnitude higher than the separate effects of these two contributions. Such results suggest that the local increase in temperature at the surface induces higher sensitivity of bacteria even towards Ag^+^ traces and leads to a remarkable synergistic effect, that could be explained assuming that membrane permeation and cell damage by Ag^+^ is promoted by local heating on cells in direct nanoscale contact with the irradiated surface^27^.

### Antimicrobial activity on bacteria directly attached to the surfaces

In addition to the planktonic experiments, we have investigated the antimicrobial effect on cells directly attached to the surfaces using SEM imaging. This aspect is particularly relevant considering that attached bacterial cells and biofilms play a critical role in infections associated with medical devices and are particularly difficult to eradicate *in vivo* ^28, 29^. The morphology of attached bacterial cells with and without irradiation was investigated using recently developed SEM fixation and staining procedures^30^, that preserve delicate biological structures at interfaces. We covered sterilised surfaces with nutrient broth containing 10^5^ CFU/mL of bacteria. The samples were protected from light, incubated at 37 °C for 24 h, and then carefully rinsed with glucose buffer. The samples were subsequently kept in glucose buffer for immediate use. For each bacterial strain, three types of samples were investigated: control (non-functionalised glass), Type II and Type III surfaces. Selected areas of the samples were irradiated for 10 s using a 785 nm laser installed in a Renishaw InVia Raman Spectrometer (shorter irradiation times were chosen to compensate for the higher power density of this laser). Adapting a previously published surface-mapping method^31^, visible-light mapping of sample morphology was performed to aid the identification of the irradiated areas (ESI SI3). After irradiation, the samples were immediately processed for SEM. The localised irradiation of selected zones of the samples allowed us to utilise the remaining non-irradiated area as a dark-control.

**Figure 5:**
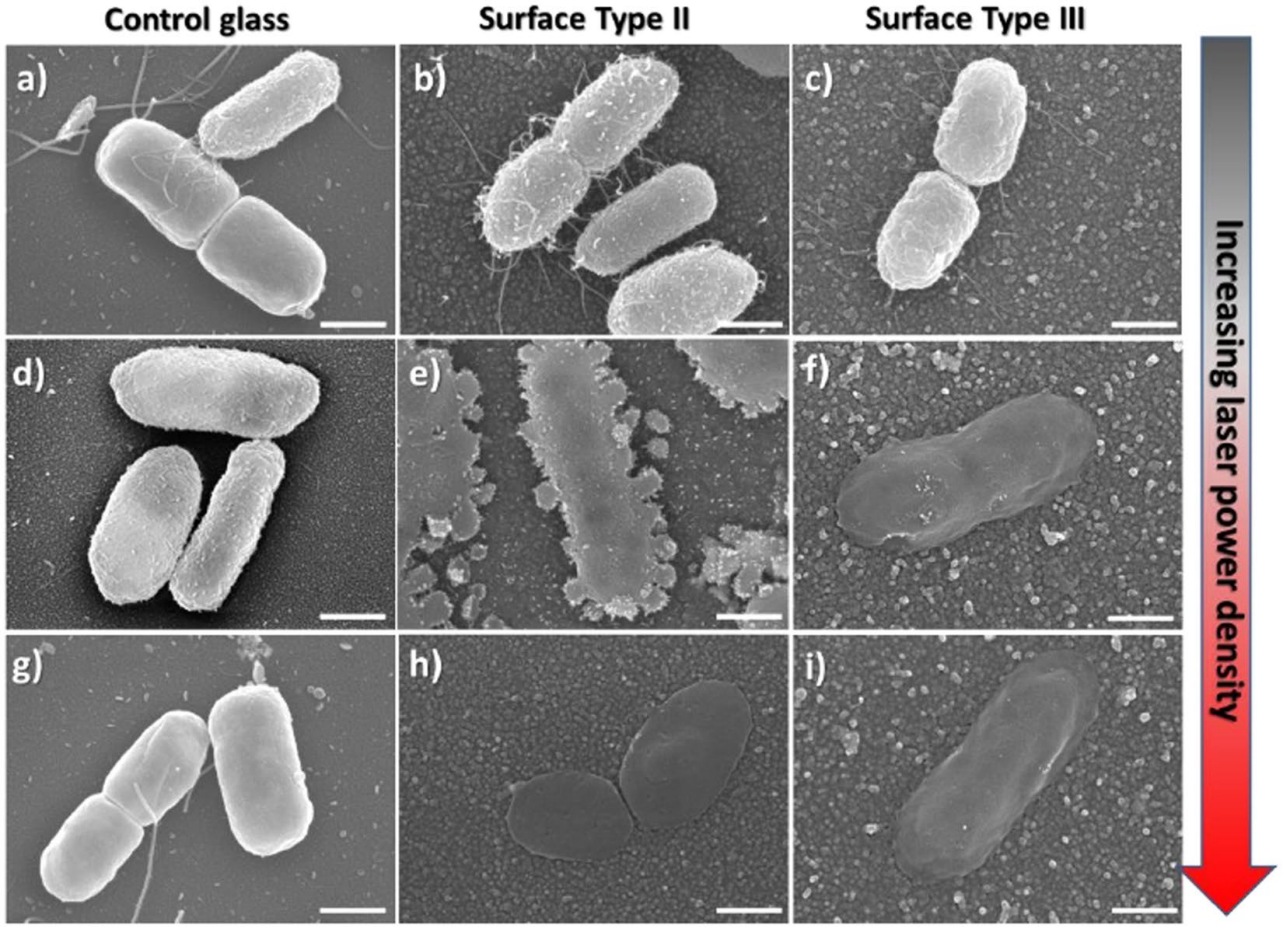
Representative SEM images showing the effect of laser irradiation on *E*. *coli* cells attached to: (by column) (**a**,**d**,**g**) Control glass; (**b**,**e**,**h**) Type II surfaces (glass|GNS|SiO_2_); (**c**,**f**,**i**) Type III surfaces (glass|GNS|SiO_2_|AgNP). Increasing power density (by row): (**a**,**b**,**c**) = non irradiated samples; (**d**,**e**,**f**) = samples irradiated with 5× objective lens; (**g**,**h**,**i**) = 20× objective lens. All scale bars are 500 nm. Large area SEM images are shown in ESI (SI14 and SI15a).

**Figure 6:**
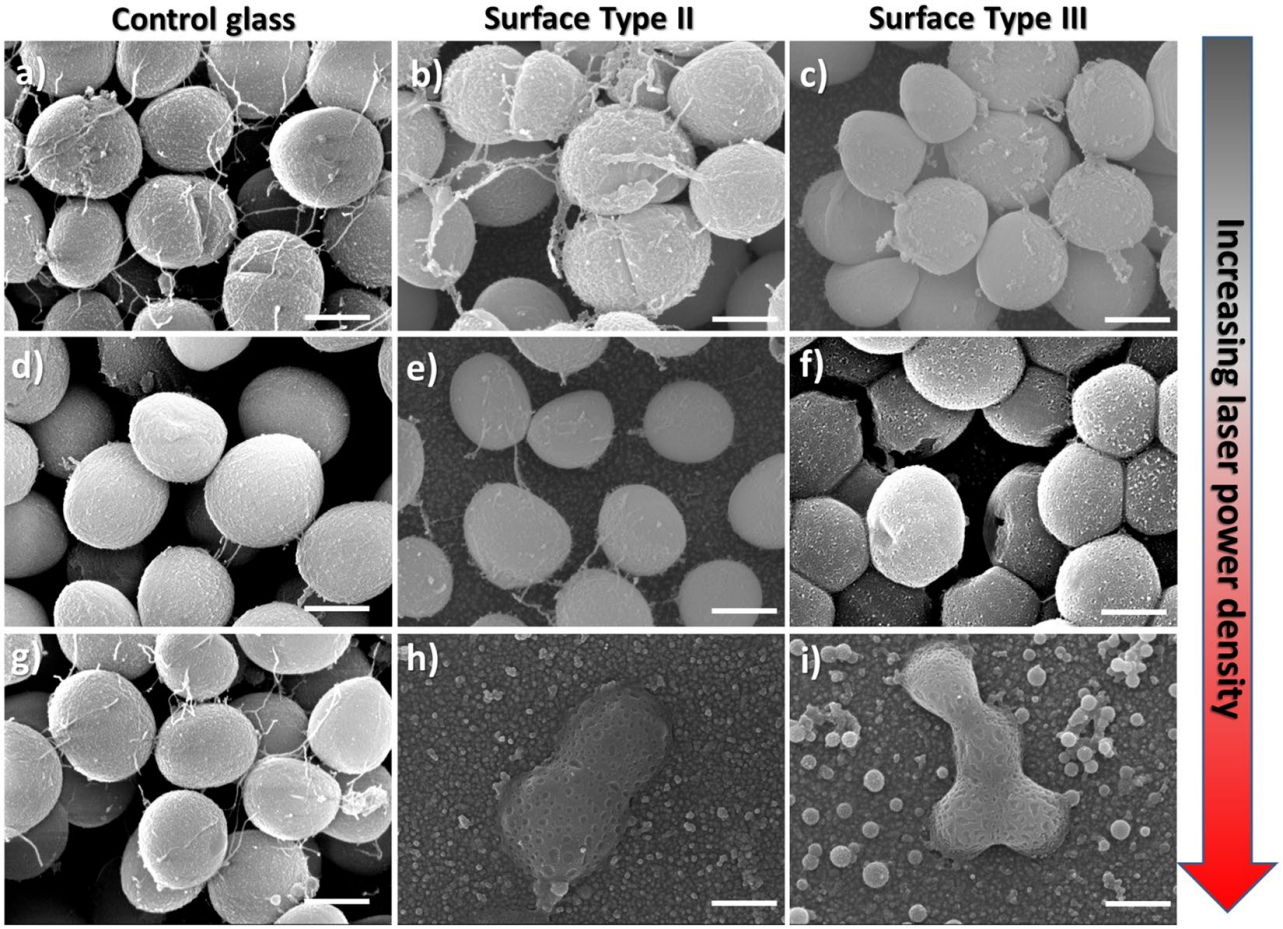
Representative SEM images showing the effect of laser irradiation on *S*. *aureus* cells attached to: (by column) (**a**,**d**,**g**) Control glass; (**b**,**e**,**h**) Type II surfaces (glass|GNS|SiO_2_); (**c**,**f**,**i**) Type III surfaces (glass|GNS|SiO_2_|AgNP). Increasing power density: (by row) (**a**,**b**,**c**) Non irradiated samples; Samples irradiated with: (**d**,**e**,**f**) 5× objective lens; (**g**,**h**,**i**) 20× objective lens. All scale bars are 500 nm. Large area SEM images are shown in ESI (SI14 and SI15b).

SEM showed that in the absence of irradiation, the morphology of the cells attached to the photoactive surfaces Type II & III was preserved (Figs 5a–c and 6a–c). We observed that the surface density of *E*. *coli* cells on the non-irradiated photoactive surfaces Type III was considerably lower than the cell density on control glass (ESI SI13), attributed to the intrinsic antimicrobial effect of AgNPs against *E*. *coli*. For *S*. *aureus*, no significant difference was observed between non-irradiated Type III and control glass surfaces (ESI SI13).

We then performed laser-irradiation experiments with increasing power densities on biofilms of *E*. *coli* and *S*. *aureus* attached to control glass surfaces (Figs 5a,d,g and 6a,d,g, respectively). On these non-functionalised glass samples, even at the highest power density used, we do not observe any adverse effect on cell morphology. This confirms that under our experimental conditions direct laser-radiation damage is negligible. When Type II surfaces containing only GNS were irradiated in the NIR, we observed a remarkable disruption of *E*. *coli* cells morphology as the laser power density increased, going from intact cells (Fig. 5b), to cells displaying a granular-like membrane disruption (Fig. 5e), to complete collapse of the cell structure (Fig. 5h). These results demonstrate that the photo-thermal effect on Type II surfaces is significantly effective on bacterial cells attached to the surface. Interestingly, the irradiation of Type III surfaces led to complete *E*. *coli* cell disruption at lower power density (Fig. 5f). Comparing Type II and Type III surfaces at intermediate power density in Fig. 5d–f we clearly observed an enhanced effect emerging from the combination of photo-switchable GNS and Ag^+^ release from AgNP on Type III samples. This enhancement is remarkable, considering that the temperature increase at the surface for both Type II and III samples is the same, irrespectively of the presence of the AgNP. This surface-located effect correlates with the synergistic antimicrobial activity observed in the planktonic state for Type III surfaces.

Similar results were obtained for *S*. *aureus* (Fig. 6), showing progressive disruption of cell morphology on Type III surfaces. In this case the intermediate state (Fig. 6d–f) was characterised by increasing number of pores on the rigid *S*. *aureus* cell wall, ultimately leading to the collapse and coalescence of neighbouring cell membranes. For this gram-positive bacterium higher power densities were required to produce complete disruption of cell morphology even on Type III surfaces (Fig. 6g–i).

## Conclusions

We have developed a new material (Type II slides) that displays the chemical and mechanical features of a glass surface, and is capable of a photothermal response upon NIR laser irradiation, due to the underlying and chemically-segregated monolayer of GNS. The versatility of such material has been exploited to graft an overlayer of AgNP, using silane/glass chemistry. These multifunctional surfaces (Type III slides) present a novel architecture with an external layer of AgNP and an underlying GNS monolayer, separated by a thin SiO_2_ coating. Within our modular design, each functionality preserves its intrinsic property in an orthogonal fashion, i.e. the GNS deliver their inherent photo-thermal activity upon near-infrared irradiation, while AgNP release similar Ag^+^ concentrations with and without light-activation. The intrinsic bactericidal action of AgNP and the photo-switchable bactericidal hyperthermia of the irradiated GNS have been examined separately and are found to be cooperating when used simultaneously, leading to a synergistic antibacterial effect. Such modular constructions, combining nano-objects with different functionalities, may pave the way towards a new generation of multimodal antimicrobial surfaces that incorporate both static and photo-switchable components. Although biocompatibility with mammalian cells (eg fibroblasts) and *in vivo* use of Type III surfaces have still to be studied, recent results indicate that surfaces bearing AgNP are not cytotoxic for human cells^32, 33^. In addition, other studies showed that non-grafted AgNP may promote the proliferation of human fibroblasts and accellerate the wound healing process^34–37^. Under this light, we suggest that implanted medical devices could benefit from the synergistically-enhanced bactericidal action of Type III surfaces, that can be activated externally by using harmless NIR laser irradiation through living tissues to tackle post-surgical infections and bacterial recolonization of internalised devices.

## Methods

The synthesis of GNS was carried out according our published procedure^19^ under the specific concentration conditions described in the ESI (SI1). The synthesis of AgNP followed our previously reported procedure^4^, with the detailed conditions described in the ESI (SI1).

### Preparation of glass slides

#### Type 0 glasses

Prior to APTES grafting, microscopy cover glass slides (21 × 26 mm) were treated with piranha solutions (3:1 sulfuric acid 95% and hydrogen peroxide 30 wt %) for 30 minutes. Then the slides were washed in water under sonication for three minutes, three times. Then the glasses were dried in an oven for 1 hour at 140 °C. The pre-treated slides were fully immersed in a solution of APTES 10% (v/v) in ethanol and allowed to react for 5 minutes at 60 °C. The amino-modified glasses were washed three times under sonication with ethanol. After this step, the samples were gently dried under N_2_ flux.

#### Type I glasses

Type 0 glass slides were fully immersed in the GNS solution for 15 hours. After immersion, the slides were washed three times in water without sonication and carefully dried in N_2_ stream. Dried samples were stored in the dark in a desiccator.

#### Type II glasses

The dried Type I samples were fully immersed in a MPTS ethanol solution 5% (v/v) for 10 minutes. After this step, the samples were washed three times with fresh ethanol and dried by N_2_ flux. For silica deposition, the slides were then dipped for 4 hour in a 1.5 wt % sodium silicate solution (after dilution of a 27 wt % SiO_2_ solution with water) kept at 90 °C. The strongly acidic cation exchanger Amberlite IR-120 was used for the adjustment of the solution pH to 8.5–9. The silica-coated substrates (Type II) were washed three times in ethanol under sonication.

#### Type III glasses

Type II slides were treated with APTES as in the initial step. The APTES-finished slides prepared were then fully immersed in the colloidal suspensions of AgNP for 15 minutes at room-temperature. After this time the slides were washed three times with water and finally dried under N_2_ flux.

All the used materials and all the physical-chemical, analytical and imaging methods and related instrumentation are described in detail in the ESI (SI1). Studies on planktonic bacteria, including ME (microbicidal effect) and TME (microbicidal effect on NIR irradiation) are described in the ESI (SI2). Studies on surface-attached bacteria, including preparation of biological samples for localised irradiation experiments, localised laser irradiation experiments, sample preparation and imaging with Scanning Electron Microscopy (SEM) are descibed in detail in the ESI (SI3).

## Electronic supplementary material

Supplementary Information
