# Supplementary material for: Modular approach for bimodal antibacterial surfaces combining photo-switchable activity and sustained biocidal release
Source: Sci Rep. 2017 Jul 12;7:5259. doi: 10.1038/s41598-017-05693-3 (PMC5507905; doi:10.1038/s41598-017-05693-3)
Supplement: Supplementary file 1 — Supplementary Information [file 41598_2017_5693_MOESM1_ESM.pdf]

SUPPORTING INFORMATION

Modular approach for bimodal antibacterial surfaces combining photo-switchable activity and sustained biocidal release

Piersandro Pallavicini, Barbara Bassi, Giuseppe Chirico, Maddalena Collini, Giacomo Dacarro, Emiliano Fratini, Pietro Grisoli, Maddalena Patrini, Laura Sironi, Angelo Taglietti, Marcel Moritz, Ioritz Sorzabal-Bellido, Arturo Susarrey-Arce, Edward Latter, Alison J. Beckett, Ian A. Prior, Rasmita Raval, Yuri A. Diaz Fernandez

SI1 . MATERIALS, SYNTHESIS, CHEMICAL METHODS AND CHARACTERIZATION

Chemicals. Gold (III) chloride trihydrate (30 wt % in HCl 99.99%), sodium borohydride (98%), L-ascorbic acid (≥99%), silver nitrate (99.8%), sodium citrate (≥99%), hydrochloric acid (≥37%), nitric acid (≥65%), sulfuric acid (95%), hydrogen peroxide (30 wt %), toluene (≥99.7%), ethanol (≥99.7%), N-Dodecyl-N,N-dimethyl-3-ammoniun-1-propansulfunate (≥99.7%), (3-aminopropyl)trimethoxy-silane (APTES), sodium silicate solution ($Na_2O(SiO_2)_{3-5}$ 27% wt % SiO_2), (3-mercaptopropyl)trimetoxysilane (MPTS), were purchased from Aldrich. Amberlite® IR120 hydrogenated was purchased from Fluka Analytical. Microscopy cover-glass slides 21x26 mm were purchased from DEL Chimica. All reagents were used as received. Water was deionized and bi-distilled. Glassware was carefully cleaned with aqua regia, and then washed three times with bi-distilled water for three minutes under sonication, before use.

Synthesis of Gold Nanostars (GSN). We followed our procedure described in A. Casu et al, *Chemistry Eur J.* (2012) *18,* 9381-9390, under the specific concentration conditions here described. *i) Seed solution.* The seeds were prepared in a vial by adding 5.0mL of LSB aqueous solution (0.2M) and 5.0mL of $HAuCl_4$ aqueous solution ($5*10^{-4}$M). Subsequently, 600µL of an ice-cooled solution of $NaBH_4$ in water (0.01M) were added to the pale yellow solution of $AuCl_4^-$ obtained in the previous step. As prepared brown-orange solution was gently hand-shaken for a couple of second; this solution is efficient for the growth procedure of gold nanostars for 180 minutes from preparation if kept cold. *ii) Growth solution.* 50mL of LSB solution in water at the same concentration chosen for the seed solution (0.2M), 1800µL of $AgNO_3$ in water (0.004M), 50 mL of aqueous $HAuCl_4$ (0.001M) and 820 µL of an aqueous L-ascorbic acid solution (0.078M) were mixed to obtain a colourless solution just after a few seconds of gentle mixing. Then120µL of seed solution were added to give a grey-blue colour liquid, the intensity of which rapidly increased. The solution was allowed to react without agitation for 1h.

Synthesis of Silver Nanoparticles. We followed the procedure described in P. Pallavicini et al, *J. Colloid Interf. Sci.* (2010) *350*, 110-116. Briefly, to 100mL of ice-cooled water were added in sequence under vigorous stirring: 1mL of $AgNO_3$ solution in water (1 wt %), after a minute 1mL of sodium citrate solution (1 wt %), after a further minute 0.75mL of a solution 0.075 wt % in $NaBH_4$ and 1 wt % in sodium citrate. After the last addition, stirring was immediately stopped to avoid coagulation. The colloidal suspension were stored in the preparation flask, and kept in the dark.

Preparation of glass slides.

*Type 0 glasses***.** Prior to APTES grafting, microscopy cover glass slides (21x26 mm) were treated with piranha solutions (3:1 sulfuric acid 95% and hydrogen peroxide 30 wt %) for 30 minutes. Then the slides were washed in water under sonication for three minutes, three times. Then the glasses were dried in an oven for 1 hour at 140°C. The pre-treated slides were fully immersed in a solution of APTES 10% (v/v) in ethanol and allowed to react for 5 minutes at 60°C. The amino-modified glasses were washed three times under sonication with ethanol. After this step, the samples were gently dried under N_2 flux.

Type I glasses. Type 0 glass slides were fully immersed in the GNS solution for 15 hours. After immersion, the slides were washed three times in water without sonication and carefully dried in N_2 stream. Dried samples were stored in the dark in a desiccator.

*Type II glasses***.** The dried type I samples were fully immersed in a MPTS ethanol solution 5% (v/v) for 10 minutes. After this step, the samples were washed three times with fresh ethanol and dried by N_2 flux. For silica deposition, the slides were then dipped for 4 hour in a 1.5 wt % sodium silicate solution (after dilution of a 27 wt % SiO_2 solution with water) kept at 90°C. The strongly acidic cation exchanger Amberlite IR-120 was used for the adjustment of the solution pH to 8.5-9. The silica-coated substrates (type II) were washed three times in ethanol under sonication.

*Type III glasses***.** The type II slides were treated again with APTES like the initial step. The APTES-finished slides prepared were then fully immersed in the colloidal suspensions of silver nanoparticles for 15 minutes at room-temperature. After this time the slides were washed three times with water and finally dried under N_2 flux.

Methods and instrumentation

*Absorption spectroscopy***.** UV-Vis-NIR absorption on functionalized slides at normal incidence was measured in air using a Varian Cary 50 UV/Vis spectrophotometer. The wavelength scan range was 300-1100 nm. The samples were placed in a special holder enabling transmission measurement of the same spot on the slide during all experimental stages.

Contact Angle. Static contact angle determinations were made with a KSV CAM200 instrument, with the water sessile drop method.

Determination of Ag and Au by ICP-OES spectroscopy. The total Ag and Au content on type I, type II and type III samples was determined by quantitatively oxidizing the silver and gold NP link on a single slide (21x26 mm coated on both sides, total coated surface 10.92 cm^2) by dipping it in 3 mL of aqua regia diluted 4:25 with bi-distilled water in a vial, and keeping it overnight on a Heidolph Promax 1020 reciprocating platform shaker. The Ag and Au content in solution were then determined by Inductively Coupled Plasma (ICP) atomic emission spectroscopy.

The release of Ag and Au versus time was followed on a set of type I, II and III slides (21x26 mm coated on both sides, total coated surface 10.92 cm^2). Each slide was immersed in 3 mL of bi-distilled water. Slides were taken off the water after 0.5, 5 and 24 h, and for type III slides also at 48, 72 and 96h. The content of Ag and Au in the 3 mL water volume was determined by ICP. Measures were repeated three times, and mean values are given. ICP data were collected with an ICP-OES OPTIMA 3000 Perkin Elmer instrument. For the determination of Ag and Au release under laser irradiation, Type III slides were cut in four portions (each 10x10mm). Each portion was immersed in 1 mL of bidistilled water inside 20x20 mm wells and irradiated with a continuous laser source tuned on 806nm for 0.5 h, using an irradiance of 0.25 W/cm^2 and a beam waist of 1.00 cm (multimode AlGaAs laser diode, L808P200, Thorlabs GmbH, power of radiation 200 mW). These are the same conditions used for the determination of the microbicidal effect on NIR Irradiation (T-ME), vide infra. Determination of the released Ag and Au followed by ICP as described.

Atomic Force Microscopy (AFM). AFM images were taken from an Auto Probe CP Research Thermomicroscopes scanning system in tapping mode using a Au coated Si probe with a typical spring constant k=1.74 Nm^{-1} (NSG03 probes from NT-MDT). Images were analysed using Image Processing 2.1 provided by Thermomicroscopes.

High-Resolution Scanning Electron Microscopy (HRSEM). Images were obtained by a ΣIGMA high-resolution scanning electron microscope (Carl Zeiss) based on the GEMINI® column which features a high brightness Schottky field emission source, beam booster, and in-lens secondary electron detector. Measurements were conducted in low acceleration potential mode (≤ 2 kV) with a working distance of about 2 mm thus allowing the use of uncoated samples.

Cross-section imaging using Scanning Electron Microscopy Back Scattering Detector (SEM-BDS). The cross sections of photoactive surfaces were imaged using a JEOL SEM 7001 instrument that provides simultaneous SEM-BDS and Secondary Electron detector images of the same sample spot. Before imaging, the samples were mechanically broken applying slight pressure with a

diamond knife on one end of the sample, and avoiding any damage of the cross-section area of interest. The cut samples were then coated on the top with 10nm thin Cr layer, to increase electrical and thermal conductivity, and minimise radiation damage. The top-coating on the flat extended surfaces of the samples did not significantly interfere with the lateral cross-section morphology and chemical composition, allowing BSD observation of electron-density contrast between gold nano-stars and SiO_2. The samples were glued on aluminium stabs using conductive carbon tape and silver paint. Imaging was performed on a 90°-tilted sample holder with short working distance to increase BSD signal. Several experimental conditions were explored, finding an optimal acceleration voltage of 15kV for high contrast images.

Transmission electron microscopy (TEM). A TEM image of the used GNS (Figure 1A, main text) was were taken on a Jeol JEM-1200 EX II instrument on 1:10 diluted GNS solution, with a 10 µL sample dropped on Nickel grids (300 mesh) coated with a Parlodion membrane

Thermograms. We employed a ThermaCAM SC 3000 (FLIR Systems; Italy) camera (320x240 microbolometers array) operating in the spectral range of 8-9µm and a numerical aperture 0.46 x 0.34. The acquisition frequency is 9 Hz with a sensitivity of 0.1°C and an accuracy of ±0.2°C. The thermocamera measured the heating of the surface of the slides. The profiles of temperature as a function of time were obtained from a ROI selected on the thermal image.

SI2 – STUDIES ON PLANKTONIC BACTERIA

*Microbicidal Effect (ME)***.** The antibacterial activity of the glasses was investigated against *Staphylococcus aureus* ATCC 6538 (Gram+) and *Escherichia coli* ATCC 10356 (Gram−). The microorganisms were grown overnight in Tryptone Soya Broth (Oxoid; Basingstoke, Hampshire, England) at 37°C. Washed cells were re-suspended in Dulbecco's PBS 10% and optical density (OD) was adjusted to 0.2, at 600 nm wavelength corresponding approximately to 1×10^8 Colony Forming Units (CFU/mL). Bacterial suspensions (10µL) were deposited on a standard microscope slide (76×26mm); subsequently the microbial suspensions were covered with a functionalized glass (24×24mm), forming a thin film between the slides that facilitated direct contact of the microorganisms with the active NP surface. The two assembled glasses were introduced in a Falcon test-tube (50 mL) containing 1 mL of PBS to maintain a damp environment. For each bacterial strain two equivalent modified glasses were prepared; the slides were maintained in

contact with the liquid films containing bacteria at room temperature for 5 and 24 h, respectively; for each time of contact an unmodified glass slide was processed in parallel as a control sample. After different times of contact, 9 mL of PBS were introduced in each Falcon test-tube under a gentle shaking to detach the assembled glass slides. Bacterial suspensions were then grown in Tryptone Soya Agar (Oxoid; Basingstoke, Hampshire, England) to count viable cells. The decimal-log reduction rate, a.k.a microbicidal effect (ME), was calculated using the formula:

$$ME = \log NC - \log NE$$

Where NC is the number of CFU/ml developed on the unmodified control glasses, and NE the number of CFU/mL counted after exposure to modified glasses. The results expressed as ME represent the average of six equivalent determinations.

Microbicidal Effect on NIR Irradiation (T-ME).

The antibacterial activity on NIR irradiation of the glasses was investigated against *S. aureus* ATCC 6538 (Gram+) and *E. coli* ATCC 10356 (Gram−). The microorganisms were grown overnight in Tryptone Soya Broth (Oxoid; Basingstoke, Hampshire, England) at 37°C. Washed cells were re-suspended in Dulbecco's PBS 10% and optical density (OD) was adjusted to 0.2, at 600 nm wavelength corresponding approximately to 1×10^8 Colony Forming Units (CFU/mL). Bacterial suspensions (10µL) were deposited in little wells (20×20mm); subsequently glass slides (24x26mm) were cut in four portion (each 10x10mm) and one of these portions was laid down upon the bacterial suspensions, forming a thin film between the slides that facilitates direct contact of the microorganisms with the active NP surface. Subsequently, the slides were irradiated with continuous laser sources tuned on 806nm for 30 minutes using an irradiance of 0.25 W/cm^2 and a beam waist of 1.00 cm (multimode AlGaAs laser diode, L808P200, Thorlabs GmbH, power of radiation 200 mW). After irradiation the inoculum was re-suspended in 1 mL of Dulbecco's PBS 10%; these bacterial suspensions were serially diluted in PBS and were then grown in Tryptone Soya Agar (Oxoid; Basingstoke, Hampshire, England) to count viable cells. The decimal-log reduction rate, microbicidal effect (T-ME), was calculated using the formula: T-ME = $\log NC_M - \log NT$ (NC_M is the number of CFU/mL developed on the modified but not irradiated control glasses; NT the number of CFU/mL counted after exposure to modified and irradiated glasses).

SI3 – Studies on surface-attached bacteria

Preparation of biological samples for localised irradiation experiments

S. aureus (DSM-346) and E. coli (ATCC-10798) were transferred from the stock to a fresh agar plate and incubated overnight at 37 ^{o}C. Three colonies were taken from the agar plate for each bacterial strains, and independently transferred to fresh nutrient broth medium (Oxoid, Ltd-Thermo Fisher) to be grown overnight in a shaking incubator (200 rpm, at 37 ^{o}C). The bacterial cell concentrations were adjusted to 10^5 colony forming units per millilitre (CFU/mL) in sterile nutrient broth. Control glass and photoactive surfaces were sterilized with 70% ethanol, dried and placed in separate wells within 24-well plates. 1mL of 10^5 CFU/mL bacterial suspensions in nutrient broth were added to each well containing the samples and then incubated at 37°C for 24h. After incubation, the supernatant was removed and the samples were carefully rinsed with 5% glucose phosphate buffer (pH = 7.59).

Localised laser irradiation experiments

For the localised laser irradiation experiments, the samples were dried at room temperature in vertical position. The drying time was less than 5min. Subsequently, the samples were transferred onto the confocal Raman microscope stage (Renishaw InVia Raman Spectrometer) and overall bright-field images were assembled, using a 20x objective lens over a 2000 x 2000 µm grid (**Figure SI3a**). Taking these bright-field images as surface maps, specific spots within each sample were irradiated for 10sec using a 785nm continuous laser (maximum power at the sample 70mW). The spots were set following straight line paths containing no more than 12 spots, separated at least by 180 µm. The separation between individual lines within the same sample was at least 1000 µm. Different relative power intensities (100%, 50%, 10%) and objective lenses (Leica 5x and Leica 20x) were combined to control the power density at the sample surface. After irradiation, a second bright-field map was recorded to ensure steady positioning of the samples during each experiment. The whole process from drying, irradiation to mapping was performed within 30minutes for each individual sample. The non-irradiated zones of the sample were considered a "dark" control. When the irradiation was completed, the samples were immediately fixed with 4% paraformaldehyde and 2.5% glutaraldehyde in 0.1 M phosphate buffer and stored at 4 °C.

Sample preparation and imaging with Scanning Electron Microscopy (SEM)

For SEM processing, the samples were fixed overnight at 4 °C with 4% paraformaldehyde and 2.5% glutaraldehyde in 0.1 M phosphate buffer. Subsequent fixation and staining steps were carried out

in water using 2% osmium tetroxide for 1h, 1% tannic acid for 1h, and 2% osmium tetroxide for 1h. Between these steps, the samples were rinsed several times with DI-water. Then, staining overnight at 4 °C with 1% uranyl acetate in water was performed. After this last staining step, the samples were rinsed with DI-water, and progressively dehydrated with increasing ethanol concentrations (*i.e.* 30%, 50%, 70%, 90% and 100%). After dehydration, the samples were critical-point dried in CO_2 (Quorum Technologies K850) and coated with 10 nm of Au/Pd (Quorum Technologies Q150T).

SEM imaging was performed using a JEOL SEM 7001 instrument (acceleration voltage 10 kV). The localisation of laser-irradiated zones was achieved adapting a mapping method published before [S. Syrenova, et al.; Nature Materials 14, 1236–1244 (2015)]. Starting with a bright-filed confocal image map (**Figure SI3a**), obtained as described above, the irradiated areas were identified at low magnification SEM (**Figure SI3b**), and then re-localised at increasing magnifications.

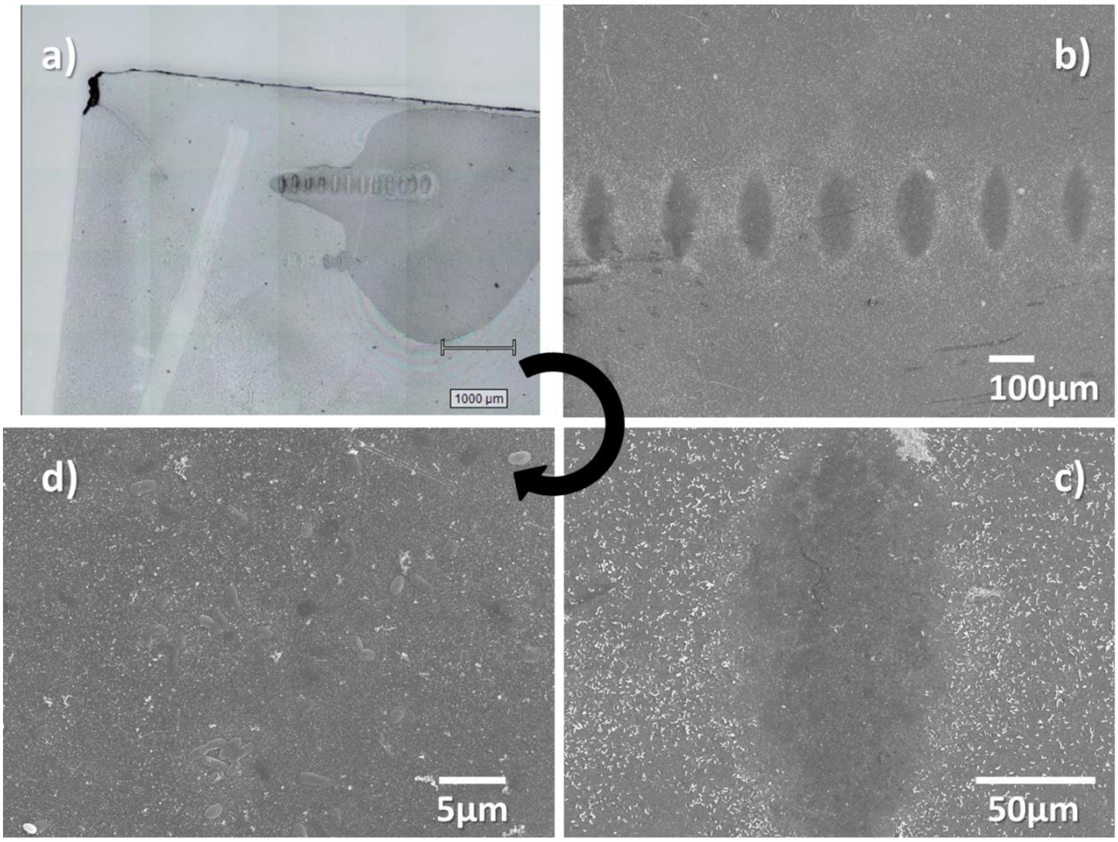

Figure SI3. Typical confocal microscope (a) and SEM images (c-d) of E. coli on Type III surfaces irradiated with 100% laser power and 5x objective lens, showing the sequential process for localisation of the irradiated spots using the mapping method based on increasing magnifications [S. Syrenova, et al.; Nature Materials 14, 1236–1244 (2015) doi:10.1038/nmat4409]. Equivalent mapping processes were performed for all the samples.

SI4 – TIME-REGULATED FORMATION OF TYPE I SLIDES. SAMPLE SELECTION CRITERIA

Type 0 slides (i.e. bearing an APTES monolayer) were dipped in a pool filled with GNS solution and removed from the pool at given times. Absorption spectra were carried out before fully oxidizing the surfaces with aqua regia to analyse both total Au and total Ag content.

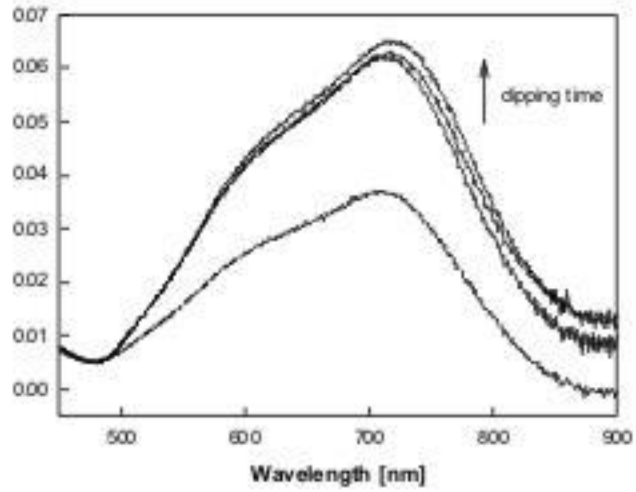

Figure SI4A. Absorption spectra (Abs vs wavelength, normalized at 480 nm) on Type I slides removed from the GNS solution jar at different times (1, 3, 15 and 24 hours)

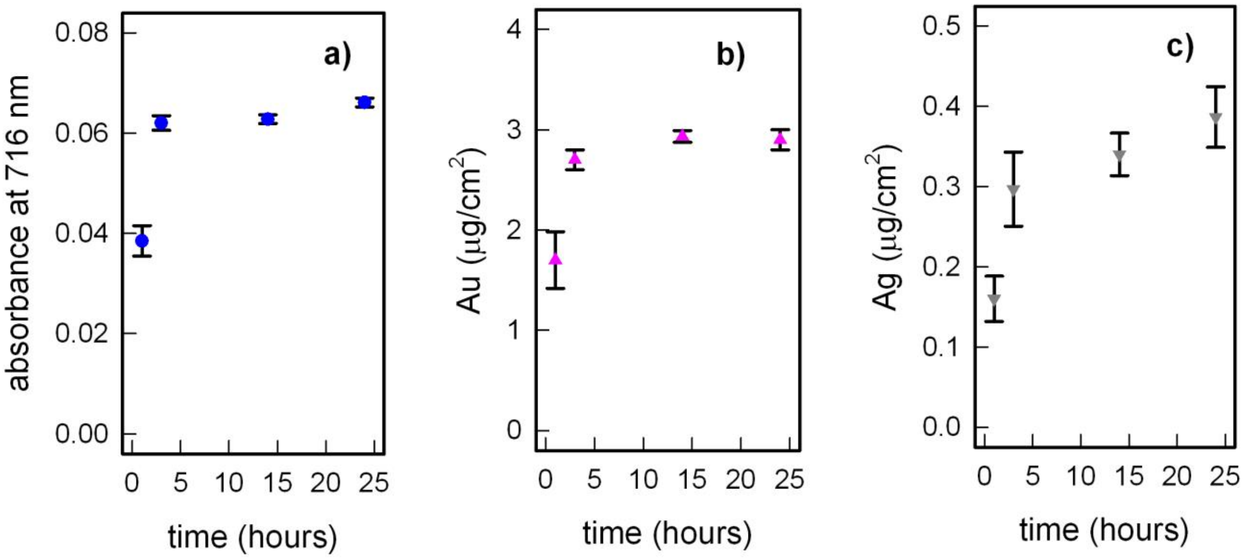

Figure SI4B. **a)** absorbance of Type I samples at λ_{max} (716 nm in the chosen glass series) as a function of immersion time; **b)** surface concentration of gold as a function of immersion time for the same series (mean values on 3 samples removed from GNS solution at the same time); **c)** surface concentration of silver as a function of immersion time for the same series (mean values on 3 samples removed from GNS solution at the same time)

Sample selection criteria. On the basis of the results of Figure SI4B we decided for a standard dipping time of 15 hours for all preparations of Type I slides. Under these conditions, preparations of glass|APTES|GNS with a 15 hours immersion time were found reproducible and the average value of surface concentration of gold was 3.0 (±0.5) μg/cm^2, based on measurements on 16 glass|APTES|GNS (Type I) samples coming from six different 15 hours preparation batches, obtained from three different colloidal syntheses.

To assure uniformity in all the preparations based on Type I slides, we decided to use for further modification only Type I samples with a normalized absorbance ranging between 0.6 and 0.8.

SI5 – Quantitative Au and Ag determination for Type I-III slides

Quantities of noble Ag and Au found in solution (ICP-OES) after treatment of samples in aqua regia, expressed as micrograms per area unit on the samples. Data are averages of least three independent samples.

sample	Au (μg/cm^2)	Ag (μg/cm^2)
Type I	3.0 (0.5)	0.32 (0.03)
Type II	2.7 (0.3)	0.30 (0.03)
Type III	2.8 (0.3)	0.9 (0.1)

From the difference between Ag data on Type III and Type II (or Type I) slides, a concentration of 0.6 μg Ag/cm^2 can be calculated for the AgNP monolayer of Type III glasses. Standard deviations for each data set are reported between brackets.

SI6 – HR SEM IMAGES OF TYPE II AND TYPE III SLIDES. SEM IMAGE OF TYPE I SLIDE

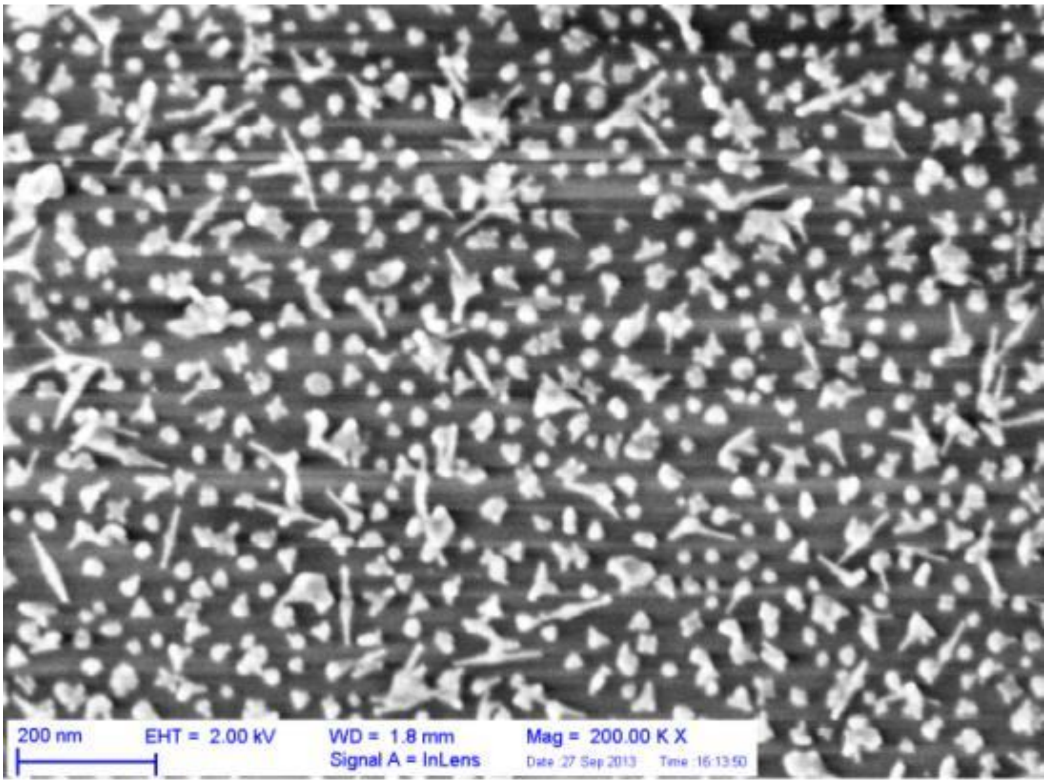

Figure SI6A – HR-SEM (200k X magnification) image of a Type II slide (Figure 1C, main text, is a detail of this image)

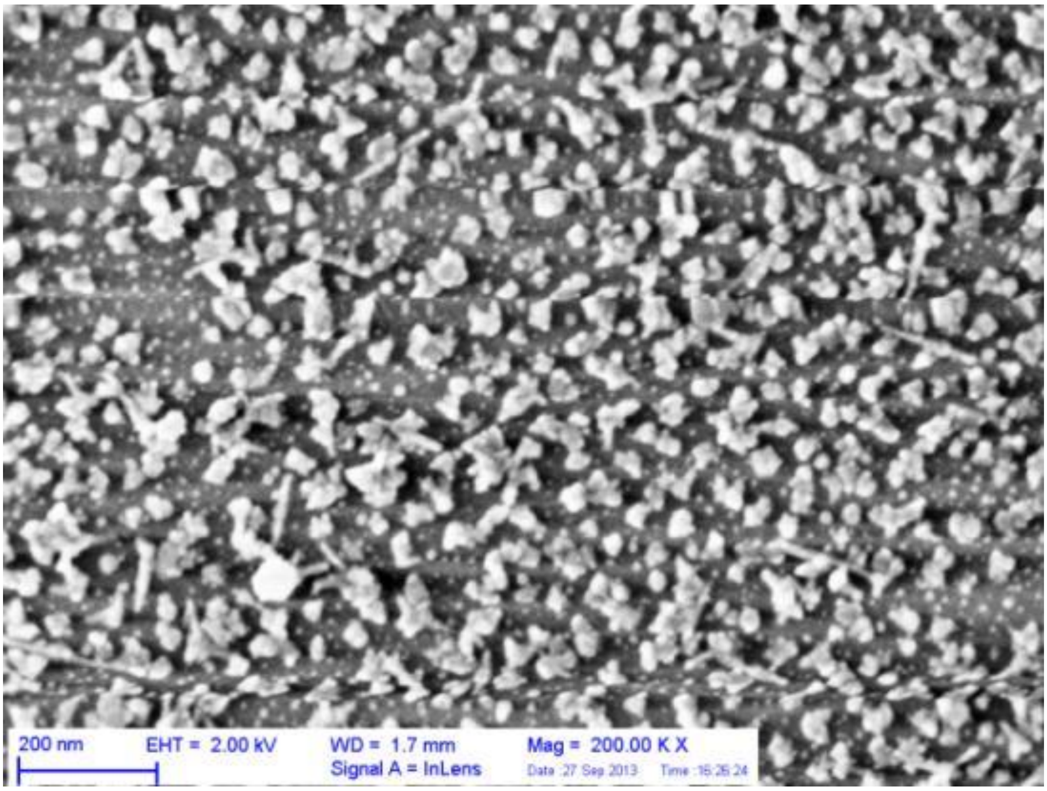

Figure SI6B – HR-SEM image (200k X magnification) of a Type III slide (Figure 1D, main text, is a detail of this image)

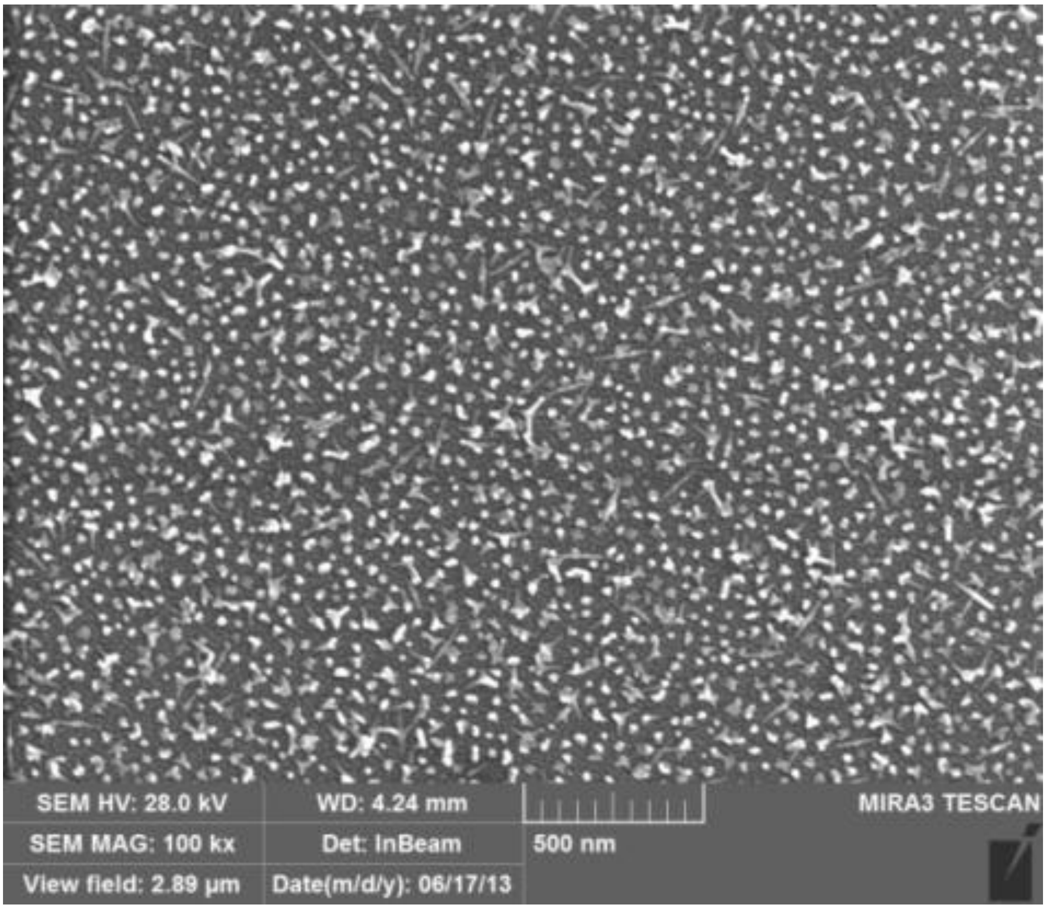

Figure SI6C – SEM image (100k X magnification) of a Type I slide

SI7 – CONTACT ANGLES (AND NUMBER OF AgNP/cm^2)

Static contact angles

Type I	37° (±4)
Type I + MPTS	66° (±4)
Type II	< 15 °
Type II + APTES, Type III	< 15°

From Type I (mainly Au surface) to Type I+MPTS surfaces there is an increase in contact angle due to the MPTS monolayer grafted on the GNS surface. Subsequent coating with SiO_2 (Type II) results in very low c.a. values due to the hydrophilicity of the SiO_2 layer prepared under these conditions (exposing SiO_m^{n-} and SiOH groups to the water interface). Type II + APTES surface gave also a c.a. < 15°, i.e. a very hydrophilic surface. APTES monolayers previously prepared on flat glass using the same technique gave c.a. of 55 ° (ref. 4, main text). In the present case, the measured low c.a. points towards the formation of a sub-monolayer of APTES upon silanization of Type II surfaces. Type III slides also gave c.a. < 15 °. A full monolayer of AgNP (see e.g. ref 4 main text) gave c.a. 32 ° therefore also in this case, a mixed surface can be hypothesise, having a sub-monolayer of AgNPs and SiO_2 still exposed at the interface.

AgNPs have an average diameter of 9 nm [ref 4 main text] and a volume of 3.81×10^{-19} cm^3 for one AgNP, corresponding to 4.00×10^{-18} g per AgNP (Ag density = 10.49 g/cm^3). According to ICP-OES, the Ag surface density is 0.60 µg/cm^3, equivalent to 1.5×10^{11} AgNP/cm^2. Using HR-SEM imagens (Figure SI6B) we have attempted to count the number of AgNP on a 200 nm x 200 nm area (4.00×10^{-10} cm^2 , ~45 NPs), focusing primarily on the SiO_2 zones, since on top of GNS the contrast was not sufficiently high to individuate the AgNPs. With this direct SEM measurement, we have estimated a surface density of 1.13 10^{11} AgNP/ cm^2, in good agreement with the ICP-OES data discussed above. Curiously, these values are slightly lower than the surface density reported before for AgNP monolayers on APTES functionalised flat glass (1.83×10^{11} [ref 4 main text]).

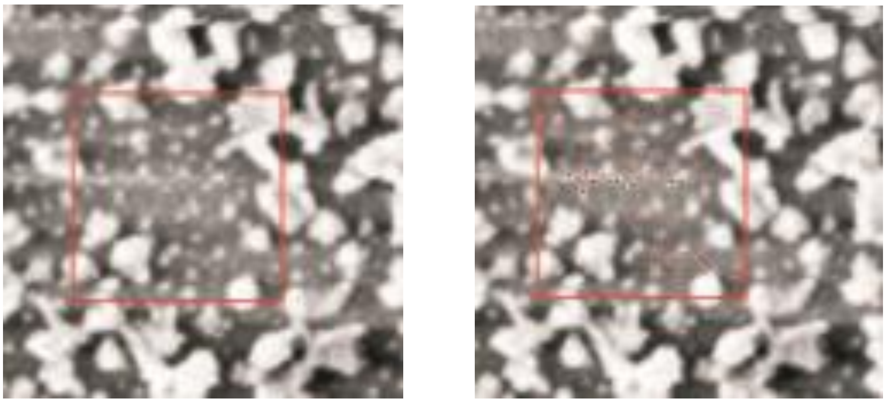

A B

Figure SI7 - **A**: Representative SEM image with selected area (red square); **B**: counted AgNP (red dots). Red squares: 200x200 nm

SI8 – AFM IMAGING

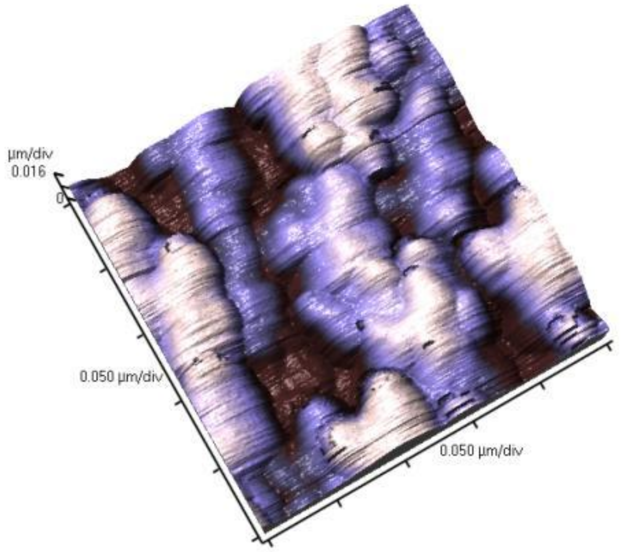

Type I (250x250 nm)

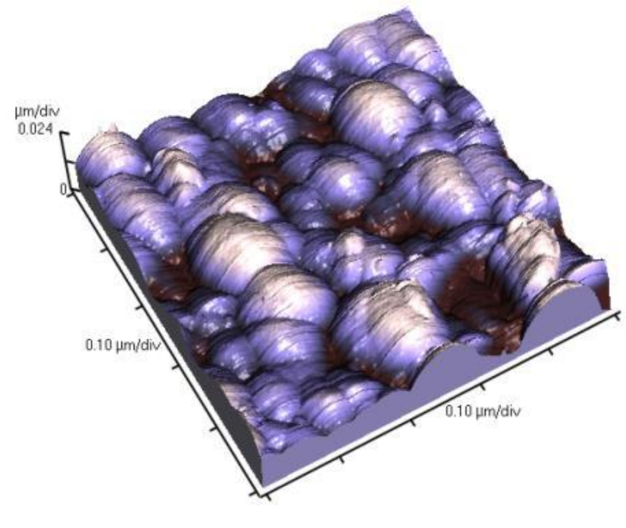

Type II (500x500nm)

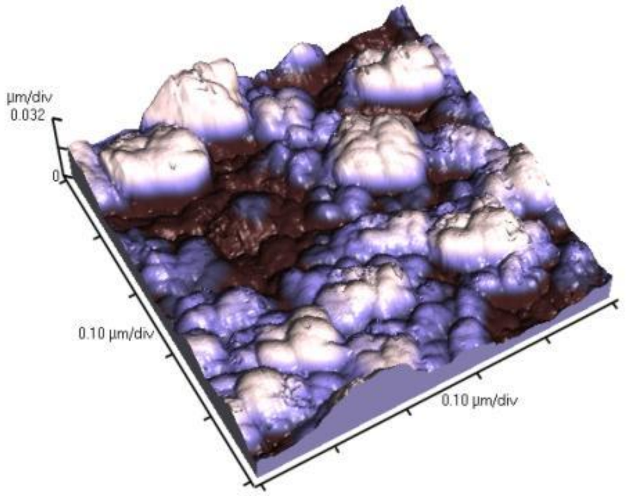

Type III (500x500 nm)

In the AFM images above, changes in morphology between Type I and Type II, and between Type II and Type III are evident. In particular, on stepping from Type I to Type II larger and smoother hills are observed. The average height of these hill-shaped objects is 20±4.1 nm (ten objects were measured with a line analysis of the image). This value can be compared with an average height of 16±4.6 nm measured on the anisotropic objects present in Type I, allowing a rough estimation of silica layer thickness of about 4-5nm. It has to be noted that in AFM imaging, the size of nano-objects is apparently larger, due to the well-known convolution effect of AFM tips on nanometre size objects [K.C. Grabar, K.R. Brown, C.D. Keating, S.J. Stranick, S.L. Tang, M.J. Natan, Anal. Chem. 69 (1997) 471].

Type III surfaces present additional, small objects, attributed to AgNP

**Line analysis heights of object (nm)**	
**Type I**	**Type II**
8.2	23.5
16.6	20.7
22.8	22
13	26.9
14.7	16.1
22.5	17.1
15.1	16.4
13.3	
13.2	
20.1	
average: 16.0 nm	average: 20.4 nm
st. dev: 4.6	st. dev: 4.1

SI9 – SEM BSD IMAGING FOR TYPE II SURFACES

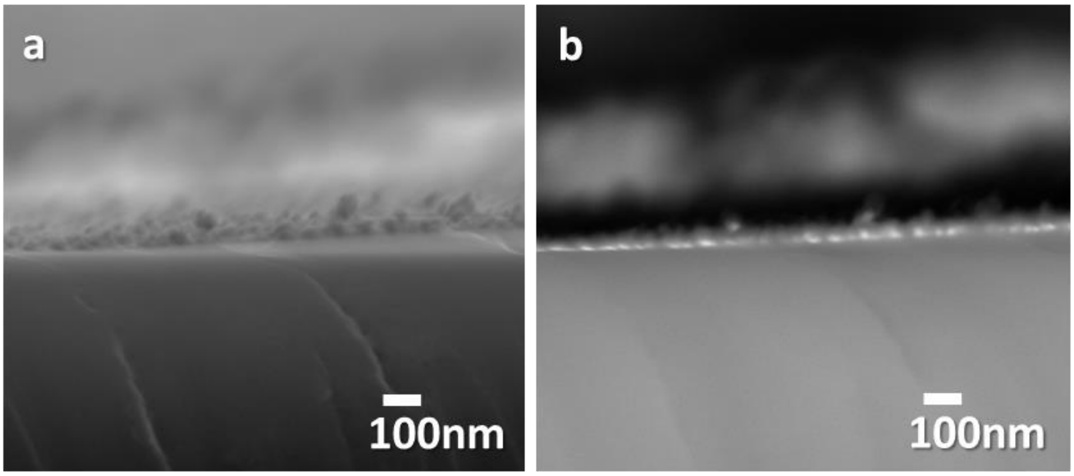

Figure SI9. Representative cross-section Scanning Electron Microscopy images of type II surfaces. a) Secondary electron detector image showing the topography of the surface. b) Back Scattering Detector (SEM-BSD) image showing electron density contrast at the cross section. The brightest spots in (b) represent the gold nano-stars on the glass surface (bottom), immersed on the SiO_2 coating. Images were taken with 15kV acceleration voltage.

Similar images for Type III surfaces are presented in the main text.

SI10 – METAL RELEASE FROM TYPE I-III SURFACES

Amount of noble metal released, after different immersion time in bi-distilled water for the three different types of samples, expressed as released micrograms per area unit.[a]

Sample (immersion time)	Au (μg/cm^2)	Ag (μg/cm^2)
**Type I** (5 hours)	-[b]	0.026 (0.02)
(24 hours)	0.006 (0.004)	0.028 (0.02)
**Type II** (5 hours)	-[b]	-[b]
(24 hours)	-[b]	-[b]
**Type III** (5 hours)[c]	-[b]	0.18 (0.03)
(24 hours)	-[b]	0.21 (0.01)
(48 hours)	-[b]	0.22 (0.03)
(72 hours)	-[b]	0.21 (0.02)
(96 hours)	-[b]	0.22 (0.01)
**Type III** (30 minutes)	-[b]	0.040 (0.02)
**Type III** (30 minutes *under laser irradiation*)	-[b]	0.050 (0.05)

[a]average values coming from at least three samples and standard deviations reported between brackets; [b]under detection limit; [c]see Figure S10 for graphical plot of Ag^+ release vs time for Type III surfaces

The release of Ag and Au versus time was followed on slides (21x26 mm) coated on both sides, total coated surface 10.92 cm^2. Each slide was immersed in 3 mL of bi-distilled water. Slides were taken off the water after 5 and 24 h. The content of Ag and Au in the 3 mL water volume was determined by ICP. Measures were repeated three times, and mean values are given. ICP data were collected with an ICP-OES OPTIMA 3000 Perkin Elmer instrument.

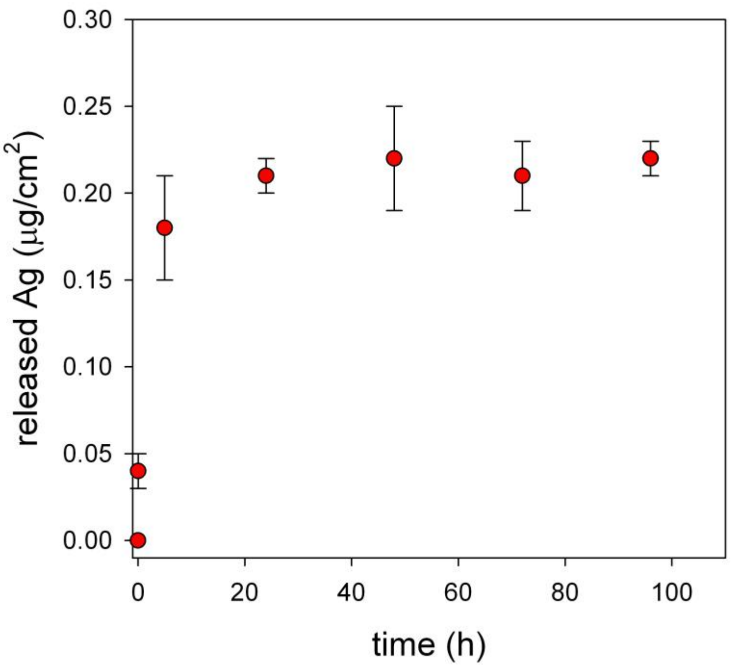

Figure S10 – Released Ag^+ vs time for Type III surface.

Data are taken from Table S10. The (0,0) point has been added to guide the eye. It has to be noticed that the release of comparable Ag^+ quantities during the first 24 h period has been altready observed for AgNP monolayers grafted on APTES (ref 5, main text). This has been attributed to the formation, in the initial period, of an Ag_2O layer on the water-exposed part of the surface of AgNPs linked to glass and placed in an aqueous environment, reaching a steady-state in which the slowly released Ag^+ ions are replaced by Ag oxidized from the bulk.

SI11 – Photo-thermal measurements

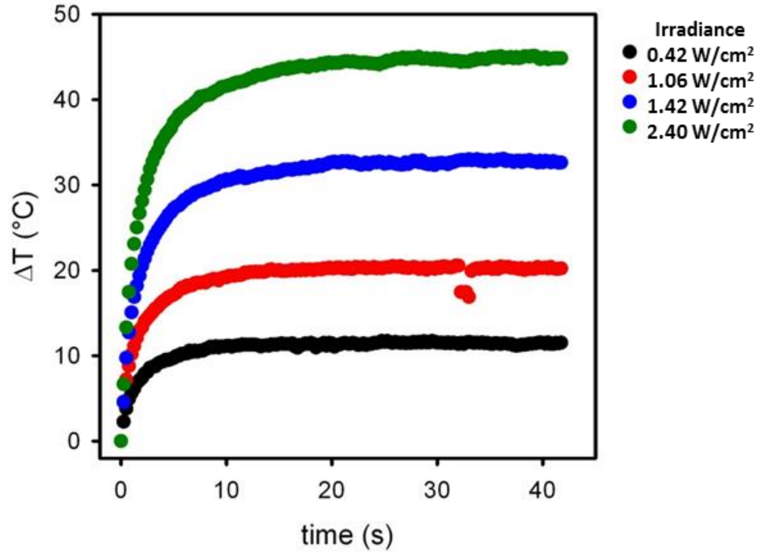

Figure S11A - Thermograms (ΔT vs time) for Type II surfaces at different irradiances (see figure for legend). ΔT are calculated from thermocamera data, i.e. from the temperature read on the slide surface at the plateau, with respect to starting T. Similar ascending/plateau profiles were observed for all Type I-III surfaces, at all irradiance (power density) values investigated.

In previous work[refSI11a,b] we have demonstrated that the thermogram profiles in Figure SI11A can be described by a double exponential growth, with two growth periods (τ_1 and τ_2) and two characteristic plateau temperatures (ΔT_1 and ΔT_2):

$$\Delta T = \Delta T_1 \left(1 - e^{-\frac{t}{\tau_1}}\right) + \Delta T_2 \left(1 - e^{-\frac{t}{\tau_2}}\right)$$

Eq. S11.1

In this expression, the overall increase of temperature at the surface after reaching stationary regime is:

$\Delta T_{max} = \Delta T_1 + \Delta T_2$. Eq. S11.2

The fitting parameters for Type II surface thermograms are presented in Table S11. It is remarkable that the two characteristic growth times are common to all the thermograms, independently of incident power densities.

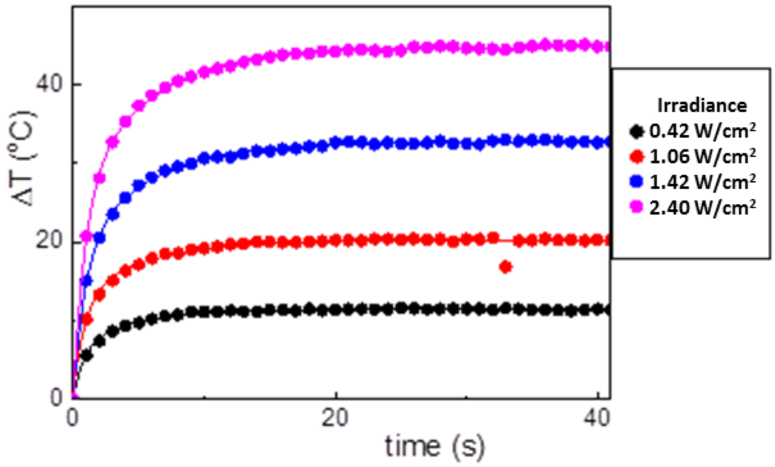

Figure S11B –Thermograms (ΔT vs time, type II) fitted to the trial function defined as in Eq. S11.1. The growth times τ_1 and τ_2 were fitted as global parameters for all the four data sets for Type II surfaces at different irradiances (see figure for legend). ΔT are calculated from thermocamera data, i.e. from the temperature read on the slide surface at the plateau, with respect to starting T. Similar ascending/plateau profiles were observed for all Type I-III surfaces, at all irradiance (power density) values investigated. The solid lines are the fit of the data to the double growth trial function S11.1.

Table S11. Best fit parameters of the temperature increase kinetics (Fig.S12B)

I [W/cm^2]	ΔT_{max}	ΔT_1	τ_1 [s]	ΔT_2	τ_2 [s]
0.42	11.5 ± 0.02	7.0 ± 0.3	0.8 ± 0.02	4.3 ± 0.2	5.3± 0.1
1.06	20.2 ± 0.03	11.7 ± 0.4	0.8 ± 0.02	7.8 ± 0.3	5.3± 0.1
1.42	32.7± 0.04	17.9± 0.4	0.8 ± 0.02	14.7 ± 0.5	5.3± 0.1
2.40	44.7± 0.05	25.0 ± 0.8	0.8 ± 0.02	20.0± 0.5	5.3± 0.1

Note: The fitting function was defined as in Eq. S11.1. The growth times τ_1 and τ_2 were fit as global parameters for all the four data sets.

These thermograms can be phenomenologically explained considering a simple physical model that accounts for photo-thermal conversion of the incident light, heat dissipation from the surface, and heat absorption at the surface. Under linear regime, considering constant incident irradiance P and constant ambient temperature T_0, the heat conservation balance leads to the following time-dependent differential equation for the temperature change at the surface:

$$\frac{\partial \Delta T}{\partial t} = \sigma \cdot P - \theta \cdot \Delta T - u(t)$$

Eq. S11.3

Where $\Delta T = T - T_0$ represents the temperature increase at the surface with respect to the surroundings; the constants σ and θ accounts for the photo-thermal conversion efficiency of the surface and the heat dissipation rate from the surface, respectively; and the time-dependent term u(t) describes the transient rate of heat absorption by the surface, before reaching the stationary regime (i.e. u(∞) = 0). At the current stage, we have direct experimental access to ΔT values by using a thermocamera, however determining u(t) is challenging. This aspect would be interesting to better understand the processes underlying the photothermal response within these systems, and is currently being object of further studies.

The plateau temperature (ΔT_{max}) in equation Eq. S11.3 is determined by the equilibrium between heat dissipation and photo-thermal conversion under stationary conditions:

$$\theta \cdot \Delta T_{max} = \sigma \cdot P$$

Eq.S11.4

That can be re-written as:

$$\Delta T_{max} = \frac{\sigma}{\theta} \cdot P$$

Eq.S11.5

This expression indicates a linear dependence between ΔT_{max} and incident power (P), in agreement with the experimental results obtained for all the photoactive surfaces studied here (FigSI11C). Moreover, Eq.S11.5 provides physical meaning for the slopes of the linear trends in Fig.SI11C, as being the ratio between the photo-thermal efficiency and the heat dissipation rate constants determining the thermogram profiles. Interestingly, the slopes in Fig SI11C for Type I, Type II, and Type III surfaces are very similar, confirming that the presence of the thin over-layers used in our modular approach is not significantly interfering with the photo-thermal behaviour of the surfaces, when irradiated by the laser in the NIR spectral region.

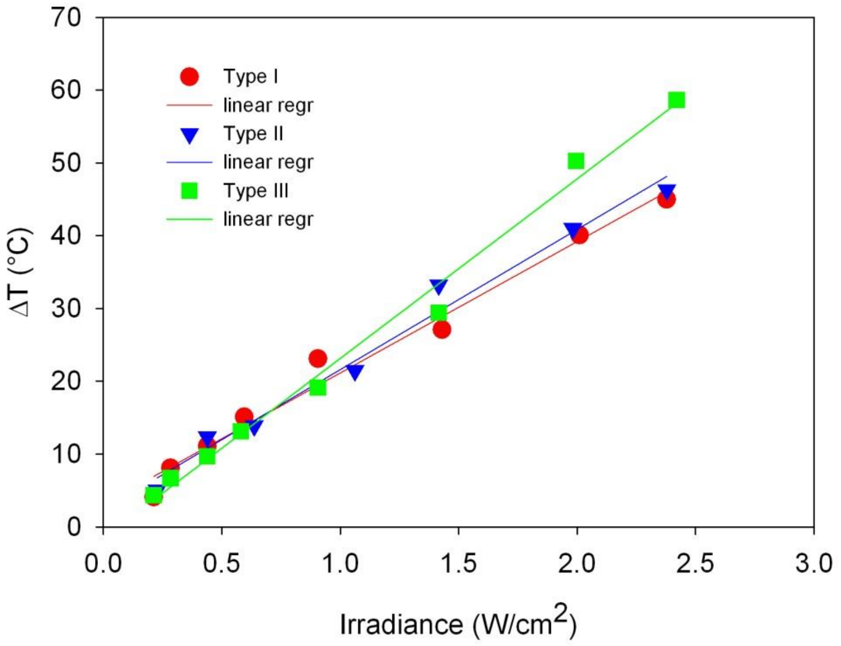

Figure S11C – Trends of T_{max} vs power density (irradiance) for Type I-III slides. Linear regression lines evidence almost superimposable trends

References: refSI11

a) Pallavicini et al. Chem. Commun., 2015, 51, 12928

b) Freddy et al. Nano Lett. 2013, 13, 2004−2010

SI12 - LASER IRRADIATION EFFECT ON BACTERIA ATTACHED TO NON-FUNCTIONALISED CONTROL GLASS (PLAIN GLASS)

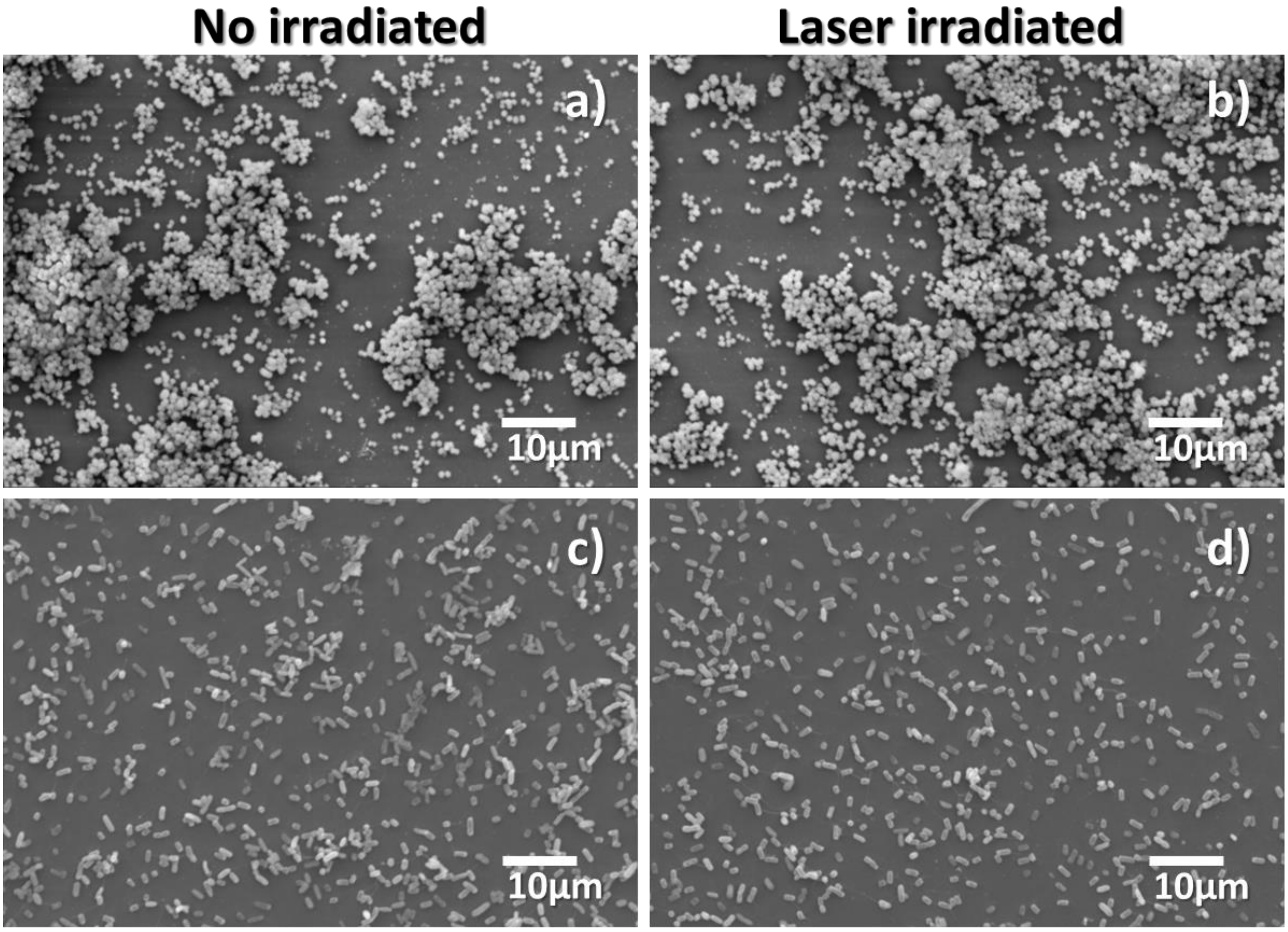

Figure SI12. Representative low-magnification SEM images of bacteria on non-functionalised control glass surfaces (plain glass) showing that laser irradiation has no effect on cell morphology or density of cells. **a)** non-irradiated *S. aureus*; **b)** laser irradiated *S. aureus*; **c)** non-irradiated *E. coli*; **d)** irradiated *E. coli*. Samples were irradiated with 100% laser power and 5x objective lens. Experimental details are described in SI1.

SI13 – INTRINSIC ANTIMICROBIAL EFFECT OF TYPE III SURFACES WITHOUT LASER IRRADIATION

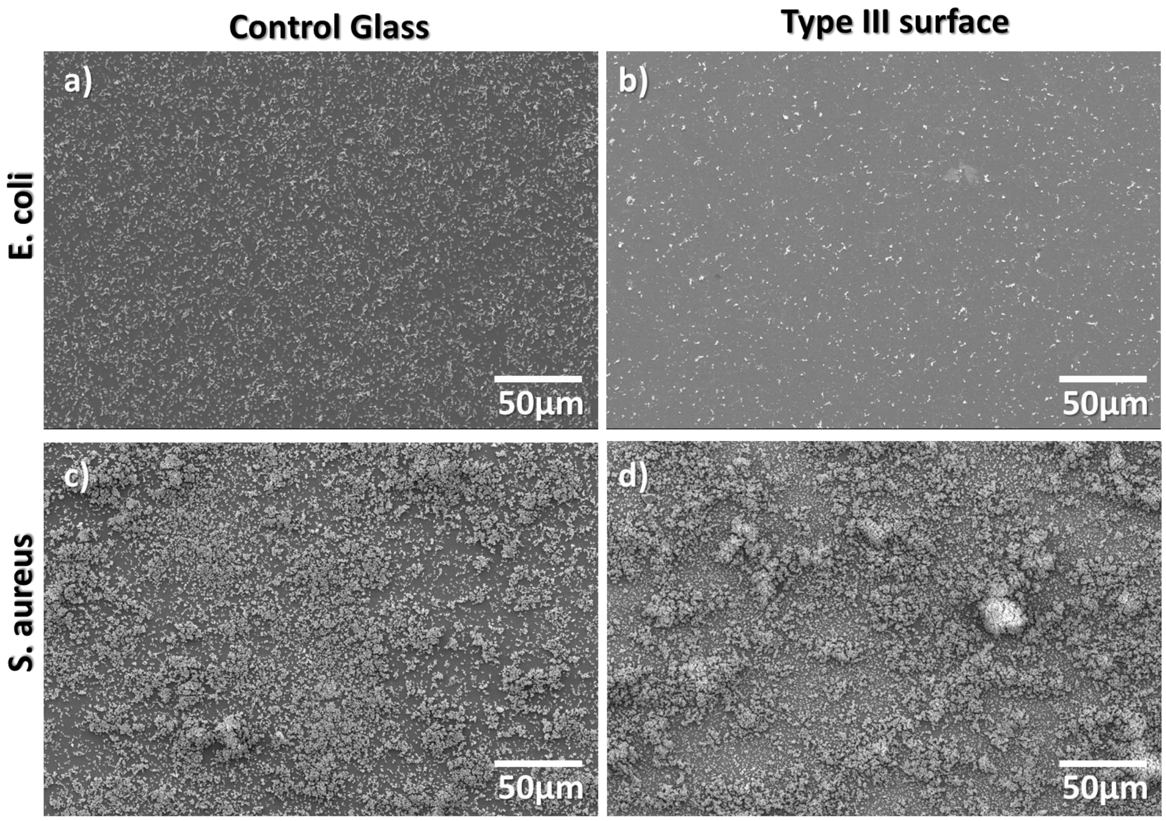

Figure SI13. Representative SEM of a) *E. coli* on non-functionalised control glass, b) *E. Coli* on Type III glass, c) *S. aureus* on non-functionalised control glass, d) *S. aureus* on Type III glass. We observed a considerable reduction in cell density for *E. coli* on Type III surfaces due to the intrinsic antibacterial activity of AgNPs. Surprisingly, this effect was not observed for *S. aureus*, probably due to the well-known higher resistance of Gram positive bacteria towards AgNPs [Amato et al., Langmuir, 2011, 27 (15), pp 9165–9173]. Moreover, in these experiments, the samples were incubated in nutrient broth for 24h (see SI1 for details); therefore we would expect the AgNPs on Type III surfaces to be capped by proteins from the rich growing media. Under these experimental conditions, the activity of AgNPs attached to the surface could be considerably reduced if compare with the non-coated AgNPs in our planktonic experiments in PBS. Similar effects have been reported before [Taglietti et al., Langmuir, 2012, 28 (21), pp 8140–8148].

SI14 – DIFFERENT BEHAVIOUR OF *E. COLI* AND *S. AUREUS* ON TYPE II SURFACES AT INTERMEDIATE LASER POWER DENSITY

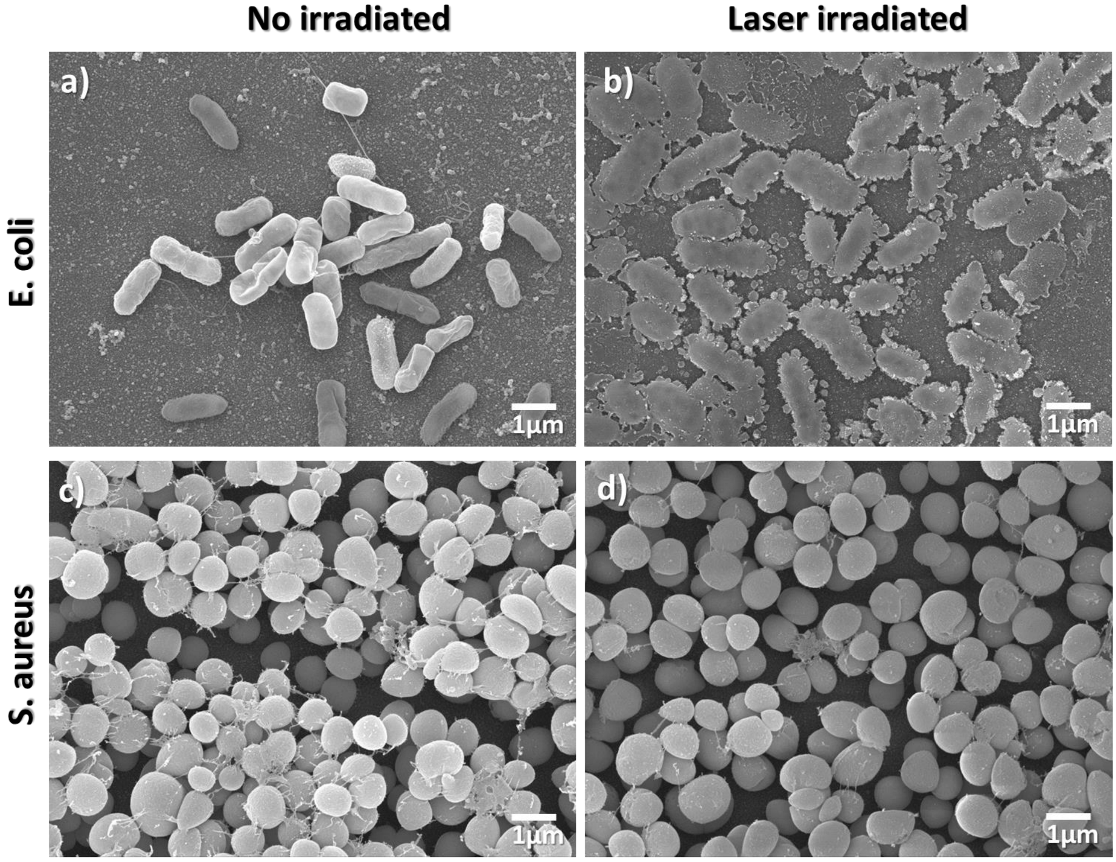

Figure SI14. Representative SEM images showing cell morphology on Type II surfaces for: a) non-irradiated *E. coli*, b) laser irradiated *E. coli*, c) non-irradiated *S. aureus*, d) laser irradiated *S. aureus*. Samples b & c were irradiated with 5x objective lens and 100% relative density power (Experimental details in SI1). Comparing these images, we can conclude that the cell architecture of *S. aureus* attached to photoactive surfaces Type II is more resistant than *E. coli* cellular structure upon laser irradiation. These results can be explained considering the thick peptidoglycan cell wall of Gram negative *S. aureus*, compared with the thin cellular membrane of *E. coli* cells.

SI15 – Large area SEM images of *E. coli* and *S. aureus* irradiated at high laser power density

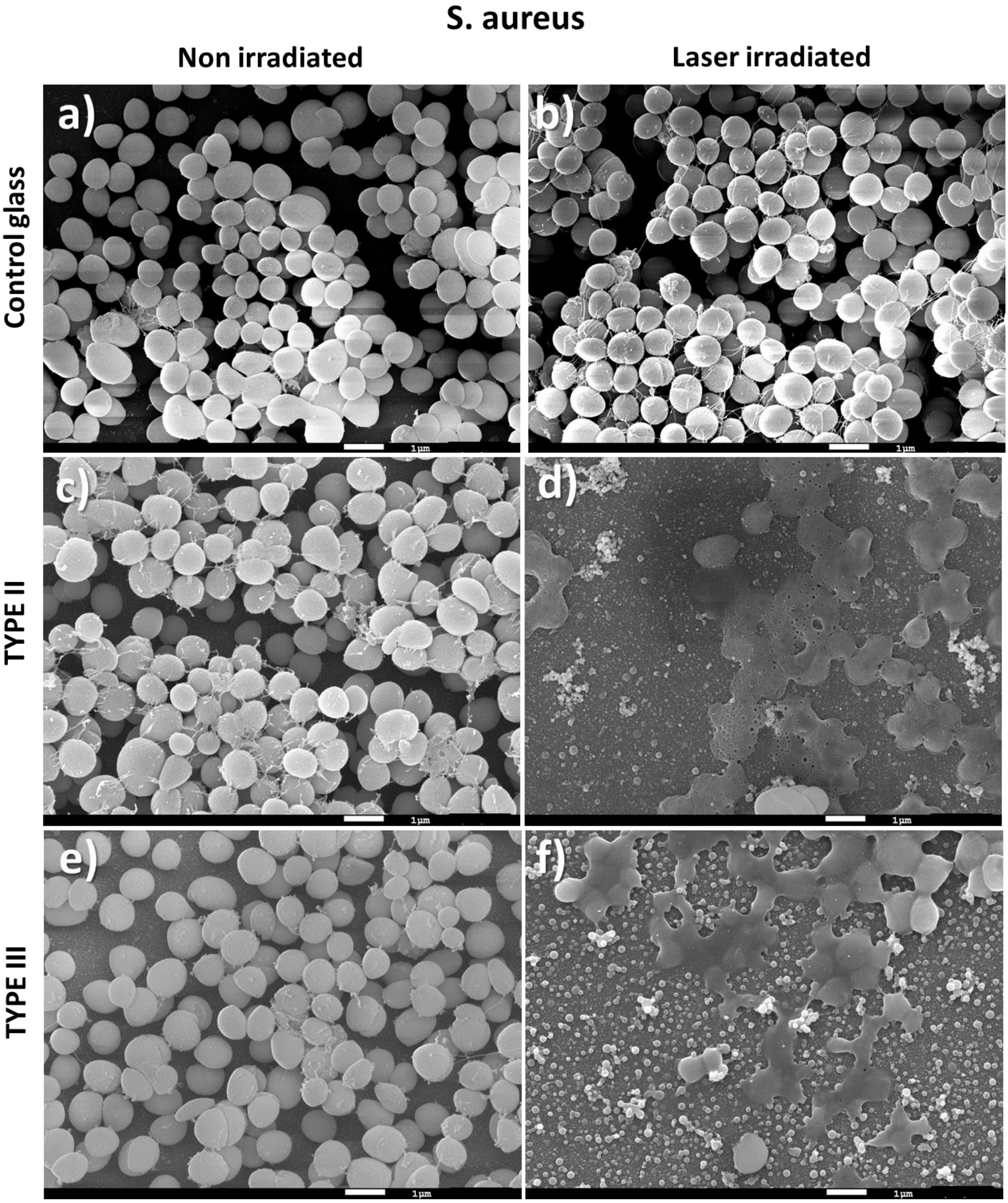

Figure SI15a. Representative large area SEM images showing effect of laser irradiation at high power on *S. aureus* cells attached to: a,b) control non-functionalised glass; c,d) Type II surfaces, e,f) Type III surfaces. Samples (b,d,f) were irradiated with 20x objective lens and 100% laser density power (Experimental details in SI1).

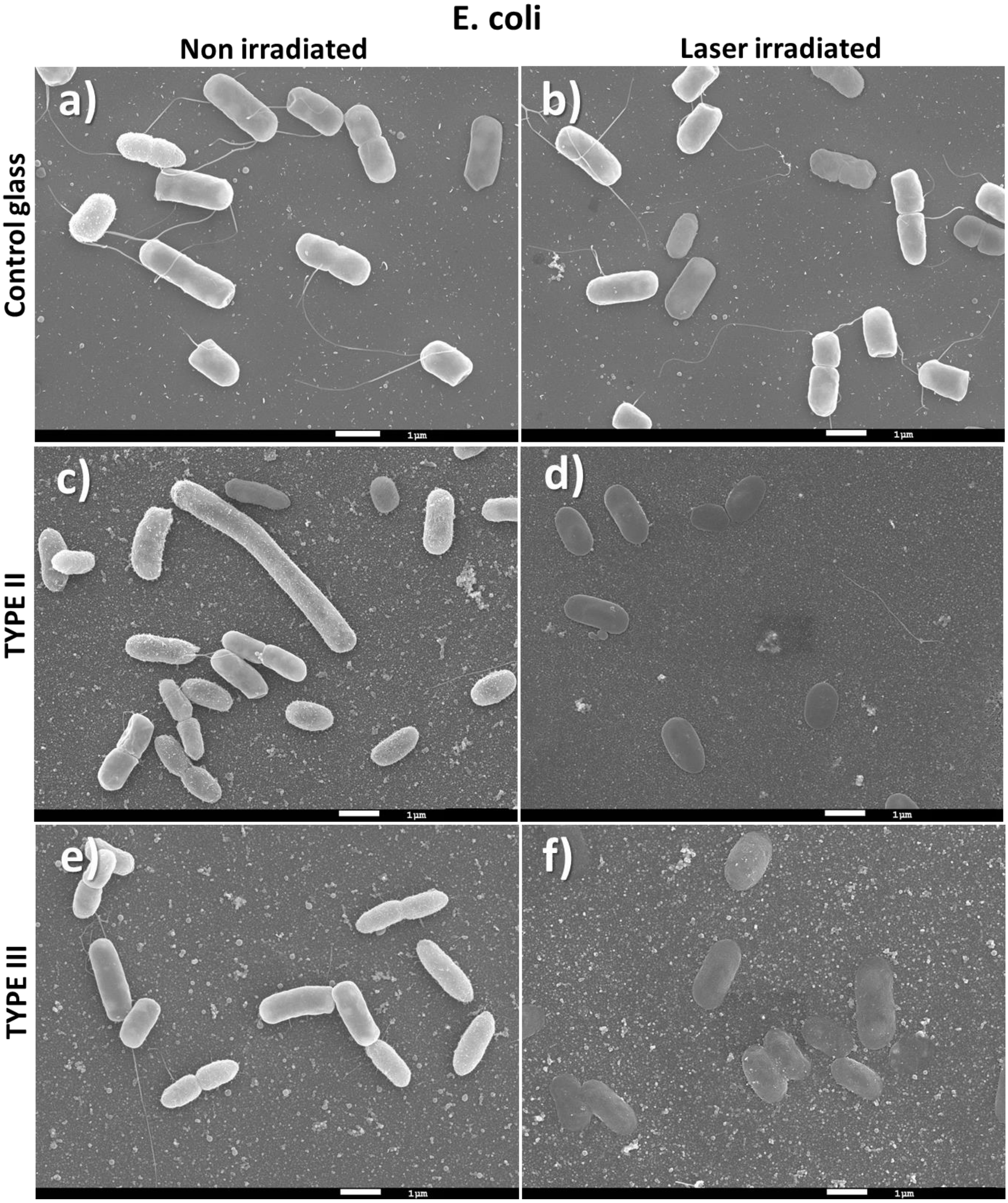

Figure SI15b. Representative large area SEM images showing effect of laser irradiation at high power on *E. coli* cells attached to: a,b) control non-functionalised glass; c,d) Type II surfaces, e,f) Type III surfaces. Samples (b,d,f) were irradiated with 20x objective lens and 100% laser density power (Experimental details in SI1).
